# The Crystal Structure of the C-Terminal Domain of the *Salmonella enterica* PduO Protein: An Old Fold with a New Heme-Binding Mode

**DOI:** 10.3389/fmicb.2016.01010

**Published:** 2016-06-28

**Authors:** Darío Ortiz de Orué Lucana, Neal Hickey, Michael Hensel, Johann P. Klare, Silvano Geremia, Tatiana Tiufiakova, Andrew E. Torda

**Affiliations:** ^1^Applied Genetics of Microorganisms, Department of Biology/Chemistry, University of OsnabrückOsnabrück, Germany; ^2^Centre of Excellence in Biocrystallography, Department of Chemical and Pharmaceutical Sciences, University of TriesteTrieste, Italy; ^3^Division of Microbiology, Department of Biology/Chemistry, University of OsnabrückOsnabrück, Germany; ^4^Department of Physics, University of OsnabrückOsnabrück, Germany; ^5^Centre for Bioinformatics, University of HamburgHamburg, Germany

**Keywords:** *Salmonella*, heme binding, cobalamin, redox co-factor, protein structure and evolution

## Abstract

The two-domain protein PduO, involved in 1,2-propanediol utilization in the pathogenic Gram-negative bacterium *Salmonella enterica* is an ATP:Cob(I)alamin adenosyltransferase, but this is a function of the N-terminal domain alone. The role of its C-terminal domain (PduOC) is, however, unknown. In this study, comparative growth assays with a set of *Salmonella* mutant strains showed that this domain is necessary for effective *in vivo* catabolism of 1,2-propanediol. It was also shown that isolated, recombinantly-expressed PduOC binds heme *in vivo*. The structure of PduOC co-crystallized with heme was solved (1.9 Å resolution) showing an octameric assembly with four heme moieities. The four heme groups are highly solvent-exposed and the heme iron is hexa-coordinated with *bis*-His ligation by histidines from different monomers. Static light scattering confirmed the octameric assembly in solution, but a mutation of the heme-coordinating histidine caused dissociation into dimers. Isothermal titration calorimetry using the PduOC apoprotein showed strong heme binding (*K*_*d*_ = 1.6 × 10^−7^ M). Biochemical experiments showed that the absence of the C-terminal domain in PduO did not affect adenosyltransferase activity *in vitro*. The evidence suggests that PduOC:heme plays an important role in the set of cobalamin transformations required for effective catabolism of 1,2-propanediol. *Salmonella* PduO is one of the rare proteins which binds the redox-active metabolites heme and cobalamin, and the heme-binding mode of the C-terminal domain differs from that in other members of this protein family.

## Introduction

*Salmonella enterica* is a food-borne Gram-negative pathogen causing severe gastroenteritis and systemic disease in animals and humans, growing under both aerobic and anaerobic conditions (Haraga et al., 2008; Hayward et al., 2015). *Salmonella* can use 1,2-propanediol as its sole energy and carbon source aerobically and anaerobically when tetrationate is available as terminal electron acceptor (Obradors et al., 1988; Price-Carter et al., 2001). It has been suggested that 1,2-propanediol is metabolized within a micro-compartment formed by a protein shell (Chowdhury et al., 2014; Bobik et al., 2015). This would help concentrate reactants and enzymes, and protect the system from side-reactions. It is certainly the case that several enzymes and potential-shell proteins are encoded in the 1,2-propanediol utilization (*pdu*) operon (Jeter, 1990).

**Figure 1:**
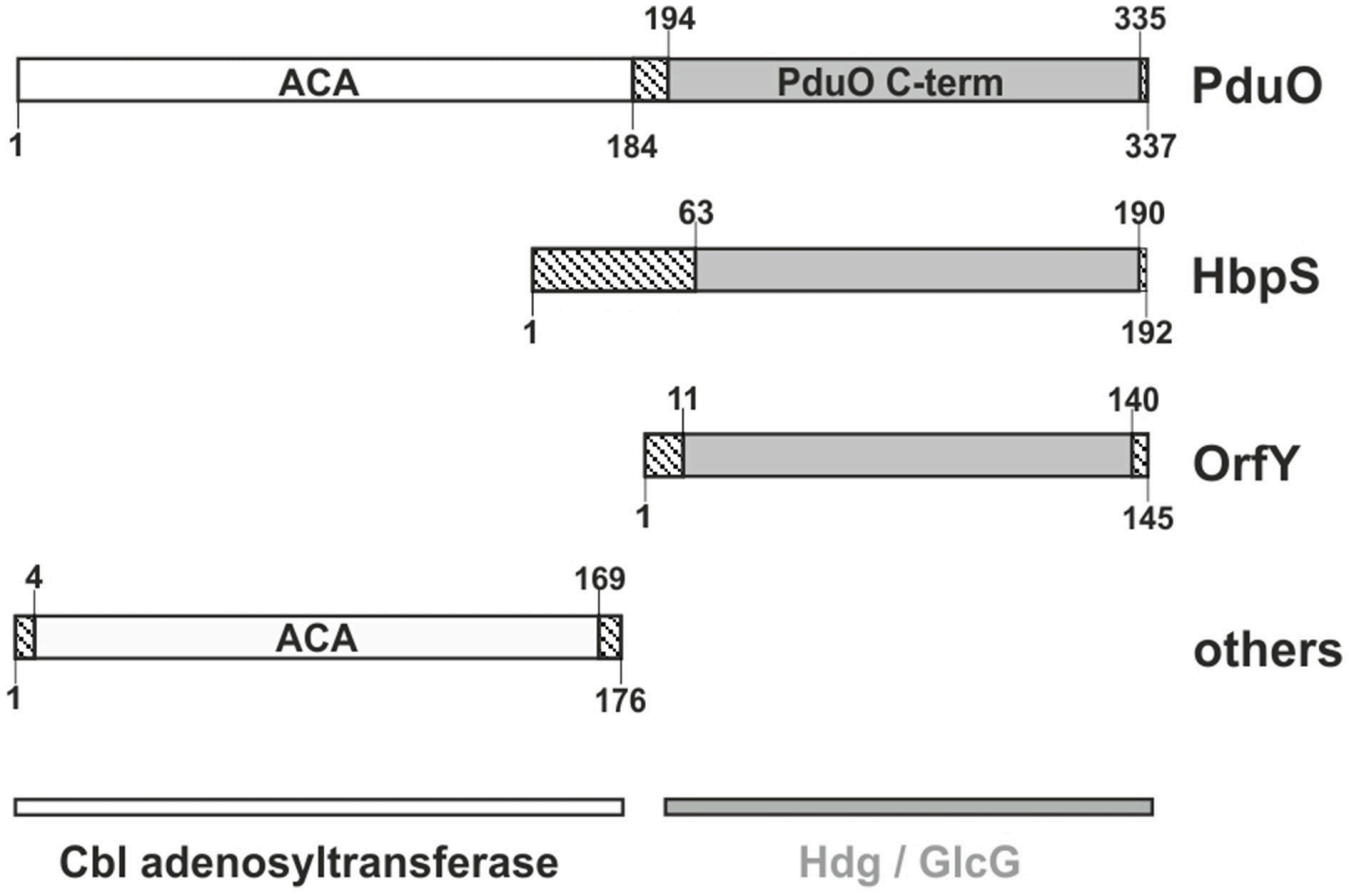
**Domain structure of PduO and homologs**. ACA refers to ATP:Cob(I)alamin adenosyltransferase. Other domain names are as given in text. Striped regions show residues outside of recognized domains. Numbers show the size (residues) in domain examples taken from *Salmonella enterica* subsp. *enterica* serovar *Typhimurium* for PduO, from *Streptomyces reticuli* for HbpS, *Klebsiella Pneumoniae* for OrfY, and a multispecies *Klebsiella* for the adenosylcobalamin transferase.

**Figure 2:**
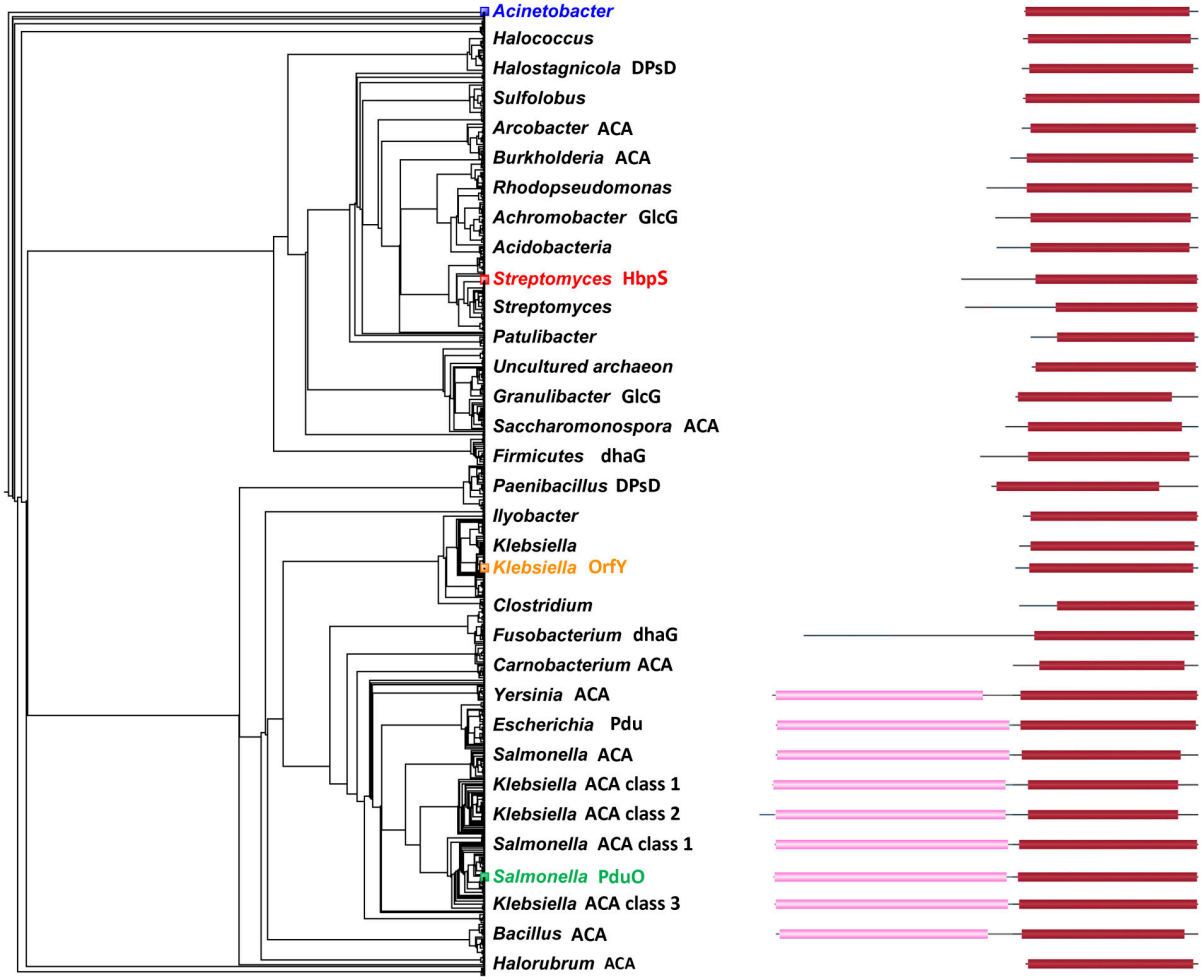
**Maximum likelihood phylogram of 1102 PduO-like sequences**. Only representative nodes are labeled. Domain structure is shown by blocks on the right with length corresponding to sequence length. Pink regions are ACA domains. Red regions are homologous to PduO C-terminus. Black lines are unannotated regions. Actinobacter is colored, as it was used to root the tree. Other colored labels show the reference/starting sequences. The domain structures come from searching with all sequences against the NCBI domain database (Marchler-Bauer et al., 2005).

Amongst its set of proteins, the *pdu* operon encodes PduO. This is an ATP:Cob(I)alamin adenosyltransferase (ACA) catalyzing the synthesis of adenosylcobalamin (AdoCbl; coenzyme B_12_). This is a co-factor of a diol dehydratase in the initial step of 1,2-propanediol degradation (Johnson et al., 2001). The *Salmonella* PduO is a two-domain protein, but only the N-terminal domain is necessary for the transferase activity (Johnson et al., 2004). This was made clear by the related protein from *Lactobacillus reuteri* (LrPduO). The LrPduO protein does not have the C-terminal domain at all, but has been co-crystallized with ATP and cob(II)alamin (Mera et al., 2009). The C-terminal domain, however, is present in numerous bacteria, both as part of the two-domain protein and with homologs found as independent proteins in even more bacteria (Figures 1, 2). Despite its ubiquity, the C-terminal domain has not been characterized. This C-terminal domain, and its structure and function are the focus of this work.

Figure 1 shows the domain structure and nomenclature for PduO and homologs. The full length two-domain protein is PduO, with its well-characterized N-terminal domain (PduON) of 197 residues. The C-terminal domain (PduOC) and homologs, however, have several names such as heme-degrading domain (Hdg), domain of unknown function (Finn et al., 2014; Galperin et al., 2015), or GlcG-like protein. Despite the name, GlcG is apparently not involved in glycolate metabolism (Pellicer et al., 1996).

Intriguingly, PduOC is related to at least two families of bacterial proteins with known structures in the profilin fold family (Murzin et al., 1995). The first family is known as OrfY (open reading frame Y) (PDB 2A2L from *Klebsiella pneumoniae*; Ramagopal et al., 2005). Maervoet et al. (2014) suggested that the protein forms a complex with a homolog of PduON (OrfW) to form a functional adenosyltransferase. This might be true, but it does not explain the function of the C-terminal domain. The second relevant family, HbpS from the soil bacterium *Streptomyces reticuli* takes its name from its heme-binding activity, with a dissociation constant *K*_*d*_ = 1 μM. It also binds aquo-cobalamin (H_2_OCbl) *in vitro* with a *K*_*d*_ = 34 μM (Ortiz de Orué Lucana et al., 2014). HpbS has a role in redox sensing (Siedenburg et al., 2012). It is an extracellular protein, but it interacts with the membrane-bound sensor kinase SenS, which, in turn, modulates the activity of the transcriptional regulator SenR. These components appear to constitute a protection mechanism against oxidative stress (Wedderhoff et al., 2013). Like OrfY, HbpS crystallizes as an octamer (PDB 3FPV; Zou et al., 2008), but there is no structure of either HbpS or OrfY co-crystallized with heme or cobalamin.

Heme and Cbl are iron- and cobalt-based tetrapyrrole macrocycles and cobalamin is found in various forms, such as methyl-, adenosyl-, cyano-, hydroxyl-, and histidyl-cobalamin. Like heme, cobalamin has a role in many different catalysts (Kräutler, 2005; Matthews, 2009; Gruber et al., 2011). Given its sequence similarity both OrfY and HbpS, one would expect this domain (PduOC) to be stable by itself, probably forming octamers. Based on the sequence similarity to HbpS, one might expect it to bind heme or some form of cobalamin.

This raises several questions. Does PduOC bind a tetrapyrrole? If so, can the protein be crystallized with the prosthetic group and does it have the octameric state ascribed to its homologs? Having identified the coordinating residues, what happens if one is replaced? Can one use the structure to exactly identify the corresponding residues in the related proteins? Finally, what does this say about the PduO C-terminal domain and homologs *in vivo*?

## Materials and methods

### Bacterial strains, plasmids, media, and cell growth conditions

*E. coli* DH5α, *E. coli* BL21 (DE3)pLysS and *S. enterica* subsp. *enterica serovar Typhimurium* strain NCTC12023 (*S. enterica*) were used. *S. enterica* wild-type and generated mutant strains are listed in Table S1. Plasmid vectors and plasmid constructs are listed in Table S2. Luria-Bertani was used as rich medium. For phenotypic characterization of the 1,2-propanediol metabolism in *S. enterica* strains, MacConkey agar base was supplemented with 1% 1,2-propanediol and 200 ng/ml CNCbl (known also as vitamin B_12_). MacConkey agar with 0.4% glucose or without an additional carbon source was also used. Plates were incubated aerobically at 37°C. Inoculation and culturing were performed as described (Johnson et al., 2001). Bacterial strains were precultured in Luria-Bertani medium, harvested, washed (4 ×) with PCN medium (lacking 1,2-propanediol and CNCbl) and used to inoculate PCN medium. Media contained 50 μg/ml carbenicillin if required to maintain plasmids. Growth assays were performed in PCN minimal medium consisting of 80 mM MOPS, pH 7.4, 4 mM Tricine, 100 μM FeCl_3_, 376 μM K_2_SO_4_, 50 mM NaCl, 1 mM K_2_HPO_4_/KH_2_PO_4_, pH 7.4, 10x micronutrients (10 nM Na_2_MoO_4_, 10 nM NaSeO_4_, 4 nM H_3_BO_3_, 300 nM CoCl_2_, 100 nM CuSO_4_, 800 nM MnCl_2_, 100 nM ZnSO_4_), 15 mM NH_4_Cl, 1 mM MgSO_4_, 10 μM CaCl_2_, 0.4% 1,2-propanediol, 1 mM each of Val, Leu, Iso, and Thr, and 200 ng/ml CNCbl (Neidhardt et al., 1974). Cultures were grown in baffled flasks at 37°C with aeration by rotation at 200 rpm.

### Isolation of DNA and transformation

Chromosomal DNA from *S. enterica* was isolated using the Blood and Tissue genomic DNA purification with the procedure for Gram-negative bacteria (Qiagen, Hilden). Plasmids were isolated from *E. coli* using a mini plasmid kit (Qiagen) and cleaved with various restriction enzymes according to the suppliers' (New England BioLabs; Thermo Scientific) instructions. Ligation was performed with T4 ligase. Purification of restricted DNA and PCR products, and extraction of DNA from gels were performed using the Qiagen gel extraction kit. Plasmids were used to transform *E. coli* DH5α and *S. enterica* by electroporation or *E. coli* BL21 (DE3)pLysS with the CaCl_2_ method.

### Generation of *Salmonella* deletion mutants

The in-frame deletions of *pduO* and *cobA* were generated using the λ Red recombineering system and primers listed in Table S3 were used to amplify the *aph* cassette of template vector pKD13 (Datsenko and Wanner, 2000). Purified linear DNA was introduced by electroporation into *S. enterica* harboring pWRG730 induced for the expression of Redα*βγ*. Recombinant strains were selected on Luria-Bertani medium plates containing 50 μg/ml of kanamycin. Proper insertion of the cassette was confirmed using primers k1-RedDel and gene-specific primers listed in Table S3. Mutant alleles were transferred to a fresh strain background by P22 transduction according to standard procedures (Maloy et al., 1996). P22 transduction was also used to combine *cobA* and *pduO* deletions. The *aph* cassette was removed by FLP-mediated recombination after electroporation with pCP20, resulting in strains remaining a scar of one FLP recombinase target (FRT) sequence (Table S1). The strain obtained was confirmed by colony PCR using DelCheck-For/Rev primers (Table S3). The deletion strains were complemented with various cloned PduO constructs and growth assays were performed.

### Cloning of *pduO* and its truncated forms for complementation studies

The low-copy plasmid pWKS30 (Wang and Kushner, 1991) was used for cloning. The overall strategy consisted of cloning the regulatory region (~400 bp) of the *pdu* operon in front of each of the investigated *pduO* genes. For that, the *pdu* regulatory region was amplified from the *S. enterica* chromosome using the primers PRegForEco and PRegRevNcoHind (Table S3). The PCR product was digested with *Eco*RI and *Hin*dIII, ligated with *Eco*RI/*Hin*dIII-cleaved pWKS30, and subsequently transformed into *E. coli* DH5α. Plasmids from selected carbenicillin-resistant transformants were sequenced. The plasmid construct carrying the desired DNA fragment was named pWKS30R (Table S2). The coding sequence of *pduO* and its truncated forms (encoding either the N-terminal or the C-terminal domain) as well as of *pduO*-His18A (see below) were obtained by *Nco*I/*Hin*dIII cleavage of the corresponding plasmids (pETPduO, pETPduON, pETPduOC, and pETPduO-H18A) described in Table S2. The *Nco*I/*Hin*dIII-cleaved *pduO* fragments were ligated with the *Nco*I/*Hin*dIII-cleaved pWKS30R and subsequently transformed into *E. coli* DH5α. The correctness of the cloning was checked by restriction and sequencing. The resulting plasmid constructs were named pWKS30RO, pWKS30RN, pWKS30RC, and pWKS30-H18A (Table S2) and used to transform *S. enterica* Δ*pduO* and *S. enterica* Δ*pduO* Δ*cobA*, respectively, by electroporation.

### Cloning and recombinant protein purification

The *pduO* region encoding the C-terminal domain was amplified from the *S. enterica* chromosome using the primers PCForNco and PORevHin (Table S3; primers were designed based on the PduO sequence EMBL: ACY88984) for cloning into the plasmid pETM11 (Zou et al., 2008). The resulting construct, pETPduOC, (Table S2) encoded PduOC fused with a His-tag and a TEV-protease cleavage site and was used to transform *E. coli* BL21 (DE3)pLysS. Protein expression was induced with 1 mM isopropyl β-D-1-thiogalactopyranoside. Protein was purified by Ni^2+^-NTA affinity chromatography. The His-tag was removed from the eluted protein with His-tag-TEV-protease in the presence of 5 mM tris(2-carboxyethyl)phosphine (TCEP, Sigma). Remaining His-tags, His-tagged-TEV protease, and uncleaved His-tag-PduOC were removed by an additional Ni^2+^-NTA affinity chromatography step. Protein purity was checked by SDS-PAGE and mass spectrometry. The concentration of purified PduOC solutions was estimated using the Bradford assay (Bradford, 1976). To ensure saturation of the protein with heme for crystallography and biochemical assays, an excess (0.5 mM) of heme B (Sigma) was added to the cell lysate from 0.5 L culture containing PduOC before the first Ni^2+^-NTA affinity chromatography.

### Crystallization, X-ray diffraction data collection, and structure determination

Ninety-six well automated crystallization trials were performed using a Tecan Freedom Evo 100. Matrix screens from Qiagen (Classics II suite) and Hampton Research (Crystal Screen, HR2-110, and Crystal Screen 2, HR2-112) were used to find crystallization conditions. In one of these trials, red/brown rhomboid-like crystals were obtained directly from the sitting drop. The crystals had dimensions of ca. 0.50 × 0.50 × 0.10 mm and grew within 3 days from a 1.0 μL drop, containing 0.5 μL of protein solution (8.5 mg/mL), and 0.5 μL of reservoir solution [0.1 M magnesium formate and 15% (w/v) PEG 3350]. The concentration of the starting protein solution was 17 mg/ml in 20 mM Tris/HCl (pH 7). This was diluted with a buffer solution of the same concentration and pH.

X-ray diffraction measurements were carried out at the ELETTRA synchrotron, Trieste (XRD1 beam-line). The crystals were cryoprotected using a 29% v/v glycerol/reservoir solution. The crystals were harvested from the mother liquor and flash-frozen at 100 K after the cryoprotection solution was added directly to the crystallization drop. Diffraction data were collected at λ = 1.0 Å on a PILATUS detector, with a detector distance of 230 mm and an exposure of 20 s per image.

Crystallographic data were processed using MOSFLM (Bailey, 1994; Winn et al., 2011) and SCALA (CCP4; Evans, 1993). A high-resolution dataset (Table 1) was obtained from a twinned crystal and the structure was solved by molecular replacement using AMORE (Navaza, 1994; Potterton et al., 2003) with the OrfY protein from *K. pneumoniae*, (PDB: 2A2L) as the starting model. REFMAC 5 was used for refinement (Murshudov et al., 1997, 2011) in a procedure consisting of starting cycles of rigid body refinement, followed by restrained refinement cycles, using non-crystallographic symmetry restraints for the protein molecules, together with direct analysis of the result and modeling of the structures by hand using wincoot (Emsley et al., 2010). Cycles of refinement were conducted until the backbone, all side chains and the solvent molecules were modeled. Numerical and statistical parameters were calculated using PROCHECK (CCP4; Laskowski et al., 1993). Pymol and Chimera were used for diagrams (Pettersen et al., 2004).

**Table 1:** **Crystallographic data on PduOC:heme**.

**PduOC:heme**	
**EXPERIMENTAL CONDITIONS**	
Beamline	ELETTRA XRD1
Wavelength (Å)	1.0
Temperature (K)	100
Detector	PILATUS 2M
**CRYSTAL PARAMETERS**	
Space Group	P21
Unit cell parameters (Å, °)	a = 71.30, b = 130.12, c = 120.75; α = β = γ 90.00
Mosaicity (°)	0.55
Twin fractions	0.204, 0.796 (-h-kl)
*%V*Solvent**	38
**DATA COLLECTION AND PROCESSING**	
No. of observations	401,563 (58,869)^a^
No. of unique reflections	152,372 (22,309)
*R*merge** (I)^b^	0.091 (0.385)
Average I/σ(I)	7.8 (2.6)
Completeness (%)	98.2 (98.8)
Multiplicity	2.6 (2.6)
**REFINEMENT STATISTICS**	
Resolution range (Å)	33.07–1.97 (2.08–1.97)
No. of reflections used	144,524
*R*factor**^c^	0.1580
*R*free**^d^	0.1856
Overall fig. of merit	0.891
**MODEL**	
No. of protein atoms	16,391
No. of heme atoms	344
No. of glycerol atoms	150
No. of water molecules	1441
Other atoms (ions)	24
Total number of atoms	18,350
**RMSD FROM IDEAL GEOMETRY**	
Bond lengths (Å)	0.019
Bond angles (°)	2.0
**RAMACHANDRAN (%)**	
Favored	99.1
Allowed	0.6
Outliers	0.3

*Experimental conditions, data collection parameters, and processing/refinement statistics*.

a *Values in parenthesis are for the highest resolution shell*.

b $R_{merge}=\frac{\sum \left(I-\langle I\rangle \right)}{\sum I}$

c $R_{factor}=\frac{\sum \left|F_{o}-F_{c}\right|}{\sum F_{o}}$ *where F_o_ and F_c_ are observed and calculated structure factors, respectively*.

d *R_free_ calculated for ca. 5% of the observed reflections*.

### Site-directed mutagenesis

The C-terminal domain of PduO contains six histidine residues at positions 15, 18, 43, 78, 96, and 146 (numbering based on the recombinantly produced Histag-free PduOC; Figure S7). Each of these His-codons was replaced with an alanine codon using a single PCR reaction with the pETPduOC plasmid construct as template and using the primers: for PduOC-H15A, PForNcoH15A and PM11RevHin; for PduOC-H18A, PM11ForNco and PRevPstH18A; for PduOC-H43A, PForPstH43A and PM11RevHin; for PduOC-H78A, PForStuH78A and PM11RevHin; for PduOC-H146A, PM11ForNco and PRevHinH146A. After PCR reactions, the amplicons were restricted with the restriction enzymes indicated in each of the primers, and then ligated with the longer fragment of pETPduOC that was previously cleaved with the corresponding restriction enzymes. PduOC-H96A was obtained with a two-step PCR technique. In the first step, pETPduOC was used as template for PCR. The reactions also contained flanking primers, PM11ForNco and PM11RevHin, as well as overlapping primers PForH96A and PRevH96A. In the second step, the PCR products (as template) and the flanking primers were used. The resulting fragments and the vector pETM11 were cut with *Nco*I and *Hin*dIII and subsequently ligated. We also replaced the codon for His18 (numbering as in PduOC) by one for Ala in the complete, intact *pduO* gene. PCR was done using the primers PM11ForNco and PRevPstH18A with the plasmid pETPduO as a template. Subsequent cloning steps were the same as to those used to obtain pETPduOC-H18A.

The resulting plasmid constructs were named pETPduOC-H15A, pETPduOC-H18A, pETPduOC-H43A, pETPduOC-H78A, pETPduOC-H96A, or pETPduOC-H146A (Table S2). To express the corresponding proteins, the constructs were used to transform *E. coli* BL21 (DE3)pLysS. The plasmid construct for the full-length *pduO* gene with the exchange His18 (nomenclature as in PduOC) to Ala was named pETPduO-H18A.

### Cloning and production of proteins for ATP:Cob(I)alamin adenosyltranferase (ACA) assays

The coding region of the full *pduO* gene was amplified from the *S. enterica* chromosome using the primers POForNco and PORevHin and the *pduO* region encoding the N-terminal domain with the primers POForNco and PNRevHin. The PCR-products were cloned into the plasmid pETM11 and the resulting plasmid constructs (Table S2), named pETPduO and pETPduON, were used to transform *E. coli* BL21 (DE3)pLysS. Synthesis of the His-tag fusion proteins in each *E. coli* BL21 (DE3) pLysS transformant was induced at an OD_600_ of 0.5 by the addition of 1 mM isopropyl β-D-1-thiogalactopyranoside to the culture medium. Cells were grown for 4 h at 37°C, harvested, washed with chilled buffer W (100 mM Tris/HCl, 150 mM NaCl, pH 8), and disrupted by ultrasonication (Branson sonifier, 5 × 10 s, with 10 s intervals) in the presence of 5 μg/ml DNaseI. Inclusion bodies were centrifuged at 30,000 g at 4°C and solubilized by ultrasonication (4 × 10 s, with 10 s rest intervals) in buffer W containing 8 M urea, 5 mM DTT, and 25 mM imidazole. Samples were then centrifuged at 30,000 g at 4°C. The supernatant was subsequently incubated with Ni^2+^-NTA agarose beads. The resin was washed with buffer W containing 4 M urea and 25 mM imidazole, and the protein eluted with buffer W containing 4 M urea and an additional 250 mM imidazole. To separate imidazole and reduce the concentration of urea, protein eluates were dialyzed against buffer W containing 2 M urea and 5 mM DTT. Because Histag-PduO and Histag-PduON precipitated rapidly in buffer lacking urea, both proteins were stored in 2 M urea. Final concentration of urea in the ACA reactions was 24 mM (see below). Attempts to cleave the Histag from the fusion proteins using the TEV protease failed (not shown), probably due to the presence of urea. We also tried to calculate the oligomeric state of both proteins by gel filtration using a solution lacking urea, but we did not record protein signals in the eluates (not shown). This was probably due to precipitation of the proteins, blocking the column matrix.

### ACA assays

The assays were performed as previously described (Johnson et al., 2001) with slight modifications. Solutions and materials were flushed with argon before use. An 83 mM titanium(III) citrate solution at pH 8.0 was prepared as described (Seefeldt and Ensign, 1994). Reaction mixtures were prepared and incubated under strictly anoxic conditions. The final volume of the reaction mixture was 1 ml containing 200 mM Tris/HCl pH 8.0, 0.4 mM ATP, 1.6 mM KH_2_PO_4_, 2.8 mM MgCl_2_, 0.05 mM HOCbl (Sigma), and 5 mM titanium(III) citrate (Sigma). Reduction of cob(III)alamin to cob(I)alamin occurred after 10 min of incubation with titanium(III) citrate at 37°C. Concentration of the proteins in the stock solution was 0.8 mg/ml. Reactions (at 37°C) were started by addition of 15 μg of each of the purified proteins as indicated above. The experiments were repeated three times and ± values show the range and not a standard error.

### Extraction of heme from PduoC:heme

PduOC apoprotein was obtained by removal of heme from the holoprotein with a protocol adapted from Hapner et al. (1968). Briefly, the pH of a 1 mg/ml holoprotein solution was adjusted to pH 1.5 with concentrated HCl at 4°C. An equal volume of chilled 2-butanone was added and the two phases were thoroughly mixed. The organic layer containing the extracted heme was discarded, and the extraction was repeated twice more. The colorless aqueous layer was exhaustively dialyzed against water. Urea was then added stepwise (in 1 M intervals) to the solution up to a concentration of 6 M. Urea was removed from the protein solution by exhaustive dialysis using buffer W (100 mM Tris/HCl, 150 mM NaCl, pH 8). The absence of heme in the apoprotein was confirmed by UV/Vis spectroscopy.

### Analysis of heme binding by PduoC

A 5 mM heme solution in dimethylsulfoxide (DMSO) was prepared as previously described (Vu et al., 2013). Before each titration, heme was diluted in titration buffer (buffer W plus 1% DMSO) to a final concentration of 20 μM. Binding was monitored by isothermal titration calorimetry (ITC) using a VP-ITC MicroCalorimeter (MicroCal; GE Healthcare, Milwaukee, WI, USA) at 25°C with a stirring speed of 307 rpm. Degassed solutions of 20 μM heme in the cell and 400 μM PduOC apoprotein (in titration buffer) in the syringe were used. Injections were applied in 30 aliquots of 10 μl each at time intervals of 180 s with an injection speed of 0.5 μl/s. The reference energy was set to 16 μcal/s. Peak integration and calculation of binding parameters were performed with Origin 7.0 (MicroCal) by fitting to the single-set-of-sites model.

Heme binding was additionally monitored spectrophotometrically using a dual-beam Specord 205 UV–Vis (Analytik, Jena) and experiments were performed in triplicate. Heme at fixed concentration (0.5 μM) was incubated with increasing concentrations of the apoprotein (0–2 μM) at 0.1 μM increments in buffer W for 30 min at 37°C. Measurements were performed using a reference cuvette containing 0.5 μM hemin.

### Cobalamin-binding assays

PduOC (10 μM) was incubated with 20 μM of either aquo-cobalamin (H_2_OCbl), methylcobalamin (MeCbl), (AdoCbl) or cyanocobalamin (CNCbl) at 37°C for 2 h. Mixtures were loaded on to a native polyacrylamide (PAA) gel that was photographed after ~30 min of electrophoresis and scanned inmediately after electrophoresis. Binding was also assessed by mixing H_2_OCbl (10 μM) with 20 μM of the apoprotein and recording UV/Vis spectra after 2 h (Ortiz de Orué Lucana et al., 2014). The H_2_OCbl-binding protein, HpbS (Ortiz de Orué Lucana et al., 2014) was used to check conditions as a positive control.

### Iron-binding assays

Iron binding was assessed with 3-(2-pyridyl)-5,6-bis(2-(5-furylsulfonic acid))-1,2,4-triazine, disodium salt (Ferene S; Sigma) staining by UV/Vis spectroscopy, and on PAA gels (Wedderhoff et al., 2013). PduOC apoprotein (20 μM) was pre-incubated with ferrous [FeCl_2_, FeSO_4_, and Fe(NH_4_)_2_(SO_4_)_2_] or ferric [FeCl_3_, Fe(ClO_4_)_3_, and FeNH_4_(SO_4_)_2_] iron salts in 50 mM MOPS (pH 7.0). Experiments were performed in triplicate.

### Fluorescence spectroscopy

Tryptophan fluorescence measurements were recorded using a Jasco FP-6500 fluorimeter. Samples contained either PduO or PduON or PduOC apoprotein (each 20 μM) in solution W plus different final concentrations (2, 3, or 4 M) of urea. Tryptophan was excited with a wavelength of 295 nm. The cell path-length was 1 cm and emission bandwidths were 5 nm. The emission spectrum was recorded from 305 to 450 nm. Each sample was measured three times and the data were averaged to obtain the shown spectra. To minimize the background, solution W plus the indicated concentrations of urea were also measured and the spectrum was subtracted from the sample spectra.

### Oligomeric state of PduoC

Multi-angle static light scattering (SLS) was used to estimate molecular weights. Wild-type PduOC and PduOC-His18Ala (H18A) mutant were purified using a MiniQ 4.5/50 PE column (GE Healthcare) before SLS measurements. The proteins were diluted in 10 mM Tris-HCl (pH 7.5), loaded onto the column, and eluted using a 38-column-volume linear gradient from 0 to 1 M NaCl in 10 mM Tris-HCl (pH 7.5). The molecular weights were estimated by passing the proteins through a Superdex 200 increase 10/300 GL size exclusion column (GE Healthcare), equilibrated with 100 mM Tris-HCl (pH 8) and 150 mM NaCl, connected to a mini-DAWN TREOS multi-angle SLS detector (Wyatt Technology) and a Shodex RI-101 refractive index detector. The absolute molecular masses were determined based on the measured light scattering and refractive index using the Astra v. 5.3.4 software (Wyatt Technology).

### Calculations

Starting sequences for searches were EMBL ACY88984.1 (UniProtKB—A0A0F6B395) for PduO from *S. enterica*, the protein databank codes 3FPV from *S. reticuli* and 2A2L from *K. pneumoniae* for HbpS and OrfY respectively. Homologs were collected with Psi-Blast in the non-redundant database, July 2015(Altschul et al., 1997).

Full length sequences were extracted and aligned with MAFFT v7.215 running in its most accurate mode with up to 100 iterations (Katoh et al., 2002). Maximum likelihood phylogenies were calculated with Raxml v8.0.19 (Stamatakis, 2014) using the LG substitution matrix (Le et al., 2008) with 200 bootstraps for the first tree and 500 bootstraps for the final tree. For the initial sequence collection, iterative searches were started with all three proteins (PduO, OrfY, HbpS) with a very conservative *e*-value for homolog acceptance (10^−20^) until one of the other three proteins was found on the list. Usually this was less than five iterations. Combining the three sets gave 1489 unique sequences. A first maximum likelihood tree was built and used to select and remove the 483 sequences most remote from the three proteins of interest. Six sequences from *Acinetobacter* were kept as an outgroup in the next calculation. The bootstrap calculations on this first tree were also used to identify the sites with the weakest confidence. To improve statistics, 14 sequences near these branch points were used to start further sequence searches and the first 50 hits from each were kept. Combining these with the sequences kept from the first tree gave a set of 1102 unique sequences used to construct the tree in Results. Sequence entropy *S*_*i*_ at site *i* in an alignment was calculated from

(1) $$S_{i}=\sum_{a\;=\;1}^{20}p_{a}\;\log_{20}\;p_{a}$$

where the summation runs over the 20 types of amino acid and *p*_*a*_ is the frequency of amino type *a* at site *i*. For the conservation plot for the full length, two-domain proteins, all 456 sequences in the non-redundant database (July 2015) were used after alignment.

Solvent accessible surface area was calculated with AREAIMOL from the CCP4 suite.

## Results

### Domain and sequence conservation in PduO-related proteins

The maximum-likelihood phylogram in Figure 2 gives the evolutionary context of this work. The labeling is sparse, but the density of the nodes shows the number of related sequences. The sequence collection used only the C-terminal domain of PduO, but the closest homologs are other two-domain proteins. Single-domain proteins, including HbpS and OrfY, are mostly more remote.

The rooted tree suggests that the HbpS family branched from other members rather early, giving it more evolutionary time to change function. We would however, not speculate on when PduO acquired the N-terminal, adenosyltransferase domain (light pink in Figure 2). There are bacteria with separate proteins which are homologous to the adenosyltransferase domain, but these are not part of the tree calculations. Given the domain movements and fusions, a simple bifurcating tree is not the full evolutionary story.

The underlying multiple sequence alignment also lets one look at residue conservation. Figure S1 shows the sequence entropy (opposite of conservation) calculated from Equation (1) using the 456 sequences most closely related to PduOC. There are conserved and variable residues in both domains, but there is no indication that the C-terminal domain is less conserved than the (N-terminal) ACA domain.

### The PduO C-terminal domain is essential for effective catabolism of 1,2-propanediol

**Figure 3:**
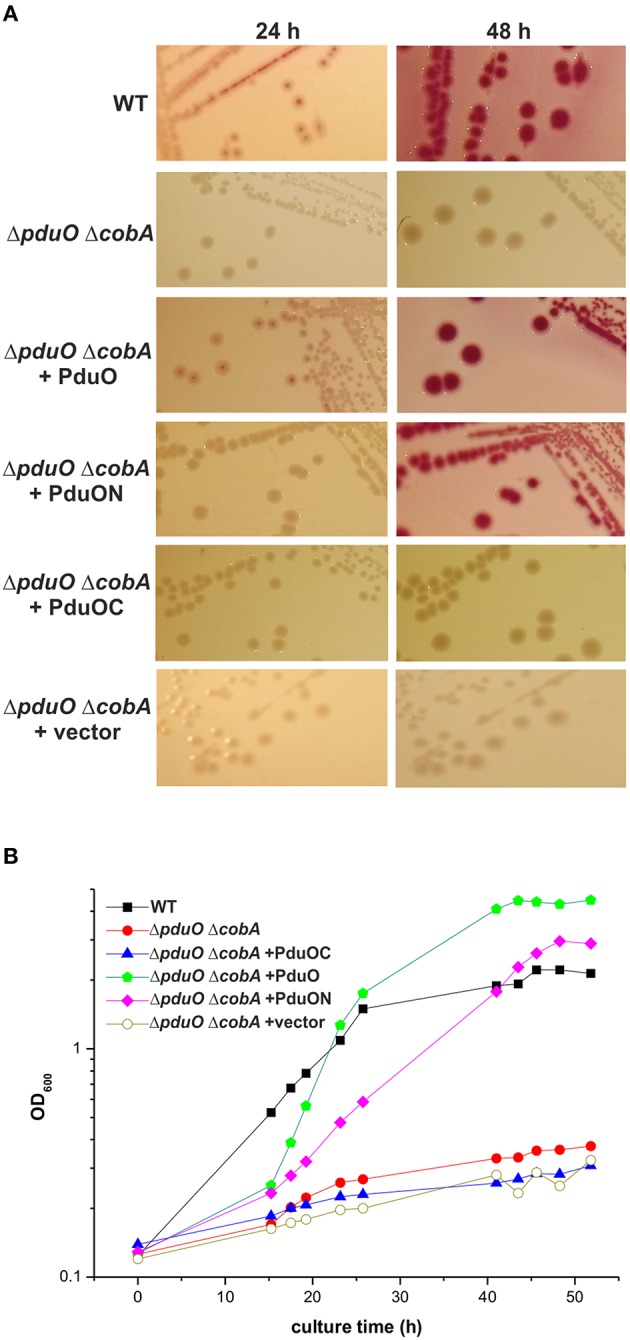
**Growth of *Salmonella* strains in 1,2-propanediol minimal medium**. Growth of the *S. enterica* WT and Δ*pduO* Δ*cobA* strains on MacConkey agar plates **(A)** or in minimal medium **(B)** both containing 1,2-propanediol and CNCbl is shown. If indicated, strains harbored plasmids expressing full length (PduO), N-terminal (PduON), or C-terminal (PduOC) moieties. The mutant strain carrying the pWKS30 plasmid (Δ*pduO* Δ*cobA*+vector) was used as a control. Cultural growth **(B)** was determined by measuring optical density at 600 nm (OD_600_) at various time points. Growth curves are representative for three independent assays with similar outcome.

An *S. enterica* in-frame *pduO* deletion mutant (Δ*pduO*) (Table S1) was used to see if the C-terminal domain of PduO (PduOC) has a physiological role. Since the *cobA* gene encodes a cob(I)alamin-adenosyltransferase that can compensate, in part, for a lack of PduO activity (Johnson et al., 2001), we also made the double deletion mutant Δ*pduO* Δ*cobA*. Plasmid constructs based on the low-copy number vector pWKS30 (Table S2) encoding PduO, PduON, or PduOC were introduced in the Δ*pduO* Δ*cobA* strain. The vector pWKS30 was also used to transform the mutant strain. MacConkey-1,2-propanediol-CNCbl indicator agar was used to detect acids produced during 1,2-propanediol degradation (Johnson et al., 2001). After 24 h of cultivation at 37°C, wild-type (WT) colonies showed a red color in the indicator medium, but Δ*pduO* Δ*cobA* as well as its pWKS30-complemented strain showed no reaction (Figure 3A). The WT phenotype was restored only by the plasmid with the full two-domain PduO. After 48 h of incubation, the WT phenotype was restored by plasmids encoding the entire PduO or PduON, but not PduOC (Figure 3A). After supplementing the medium with glucose, all strains grew with intense glucose fermentation and acid production, while growth on MacConkey base agar without a carbon source did not result in indicator reactions by any of the strains (not shown).

This was analyzed in more detail using *Salmonella* strains (Table S1) in PCN minimal medium containing 1,2-propanediol and CNCbl. The Δ*pduO* Δ*cobA* mutant had a very low growth rate compared to the WT and only achieved two-fold increase in cell mass over 42 h of incubation (Figure 3B). Complementation with full-length *pduO* restored the growth of the Δ*pduO* Δ*cobA* mutant strain, and the final optical density of the complemented strain was even higher than for the WT strain. The Δ*pduO* Δ*cobA* mutant complemented with *pduOC* as well as with pWKS30 was unable to grow (Figure 3B). In contrast, the Δ*pduO* Δ*cobA* mutant complemented with *pduON* grew to a final optical density between wild-type and the mutant strain complemented with full length *pduO*, but the growth was delayed. This indicates impaired 1,2-propanediol utilization by *Salmonella* in the absence of *pduOC*.

The data suggest that not only the N-terminal domain, but also the C-terminal domain of PduO is important for effective catabolism of 1,2-propanediol.

### PduoC is a strong heme binder and His18 is essential for binding

**Figure 4:**
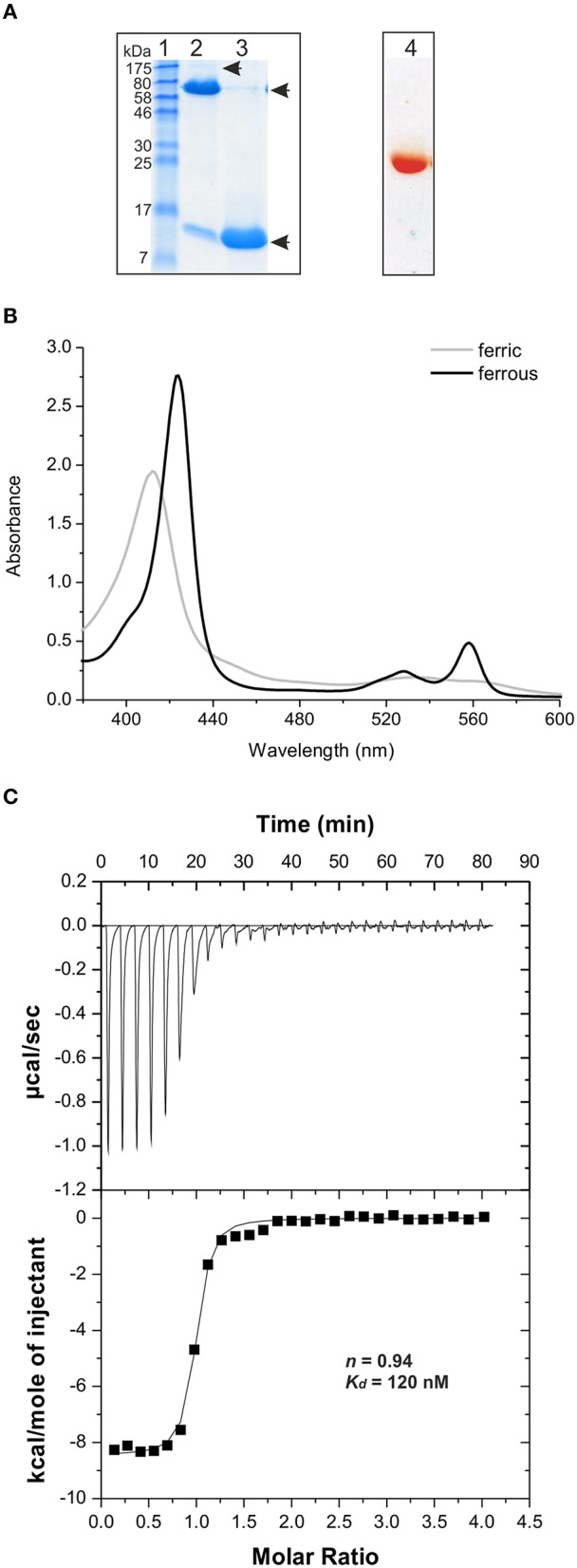
**Analysis of heme binding by PduOC. (A)** An unboiled (lane 2) and boiled (lane 3) aliquot (15 μg) of the isolated PduOC protein were analyzed by SDS-PAGE. The proteins were stained using PageBlue. The molecular weigth (in kDa) of protein markers (lane 1) is indicated. The arrows indicate the observed protein bands. The PduOC aliquot was aditionally analyzed by native PAGE (lane 4). After electrophoresis the native gel was scanned. **(B)** Isolated PduOC (50 μM) was analyzed by UV/Vis spectroscopy. The spectrum of the ferric (gray) and ferrous (black) form of PduOC-bound heme are indicated. **(C)** Binding isotherm for PduOC apoprotein titrated into heme. The apoprotein was injected stepwise (30 × 10 μl) into a cell containing heme (20 μM) at 25°C. Upper panel: Heat evolved upon injection of the apoprotein into heme as a function of injection order. Lower panel: Integrated heats of reaction plotted against the molar ratio of PduOC apoprotein to heme concentration. The best fit to the data according to a model assuming a single set of identical sites is indicated by a solid line. The calculated stoichiometry value *n* as well as the dissociation constant *K*_*d*_ are indicated.

To investigate the biochemistry of the C-terminal domain, recombinant PduOC was produced in *E. coli*. Harvesting the isopropyl β-D-1-thiogalactopyranoside-induced *E. coli* cells producing PduOC (Figure S2A) gave a strongly red/brownish-colored pellet (Figure S2B). His-tag-free PduOC was subsequently isolated. The protein eluates were red/brownish-colored (not shown). We firstly analyzed the protein by SDS PAGE. Interestingly, the unboiled protein migrates predominantly as ~60 kDa protein (Figure 4A, lane 2). There is a trace of a larger oligomeric form as well as a monomeric form (~15 kDa). The boiled sample migrates predominantly as a ~15 kDa protein, although traces of the other protein forms can also be seen (Figure 4A, lane 3). An aliquot of the protein eluate was subsequently analyzed by native PAGE. The protein migrates on the gel as a red/brownish-colored protein band (Figure 4A, lane 4). From the native PAA gel, we cannot, however, estimate its molecular weight.

The red/brownish colored protein eluate was analyzed by UV/Vis spectroscopy, giving a spectrum typical of protein-bound ferric heme (Izadi-Pruneyre et al., 2006), with a Soret band at 411 nm and α and β bands at 560 and 533 nm, respectively (Figure 4B). Treatment with the reducing agent, sodium dithionite shifted the peaks toward 424, 528, and 558 nm, corresponding to the ferrous form of protein-bound heme (Figure 4B). These data show that PduOC forms SDS-resistant oligomers, binds heme *in vivo* and is redox-active.

To identify the heme type bound at PduOC, the protein eluate was analyzed by Matrix Assisted Laser Desorption/Ionization Time of Flight (MALDI-TOF) mass spectrometry. In addition to the protein signals (not shown), another signal with a molecular mass of 615.5 ± 0.2 Da (not shown) was recorded. This corresponds to heme type B with a molecular mass of 616 Da (Li et al., 1993).

PduOC has six His residues (positions 15, 18, 43, 78, 96, and 146) which were potential heme-coordinating residues. Each was replaced by an alanine. Like the *E. coli* cells producing WT PduOC, H15A or H43A or H78A or H96A or H146A mutant cells gave brownish/red pellets (Figure S2). However, the cells and the cell pellet of the *E. coli* transformant producing PduOC-H18A did not display the red/brownish color (Figure S2). The UV/Vis spectrum of the isolated PduOC-H18A lacked the Soret band at 411 nm (not shown). These data strongly suggest that His18 is involved in heme binding.

PduOC:heme affinity was measured by isothermic titration calorimetry (ITC). Heme-free PduOC (apoprotein) was obtained by extraction of heme from the holoprotein. ITC with 20 μM heme showed that the binding to PduOC is exothermic with a dissociation constant *K*_*d*_ = 1.2 × 10^−7^ M (Figure 4C). The PduOC monomer to heme stoichiometry was calculated to be ~1. Given that heme at high concentrations forms dimers in solution (de Villiers et al., 2007), we repeated the measurements with 10 μM heme, giving a monomer:heme stoichiometry of ~1.5 and *K*_*d*_ ≈ 2 × 10^−7^ M (Figure S3A). Because the signals were close to the detection limit, no ITC experiments were attempted with lower heme concentrations.

To more precisely calculate the stoichiometry of interaction, we used the spectrophotometric heme titration method of Yonetani (1967) with slight modifications. Heme at fixed concentration (0.5 μM) was incubated with increasing concentrations of the apoprotein (0–2 μM at 0.1 μM increments) in titration buffer at 25°C for 30 min, and the samples were measured by UV/Vis spectroscopy. The plot showing the difference absorbance (ΔA) at 411 nm vs. protein concentration indicates a PduOC monomer:heme stoichiometry of ~2:1 (Figure S3B).

We conclude that PduOC is a strong heme binder [*K*_*d*_ = (1.6 ± 0.4) × 10^−7^ M], His18 is directly involved in heme binding, and the stoichiometry of interaction is most likely 2:1.

The heme-binding of full-length PduO and its N-terminal domain (PduON) was then checked. The pellet of IPTG-induced *E. coli* carrying full-length PduO was red/brownish, but the corresponding pellet from bacteria with PduON was not (Figure S4A). This indicates that the full-length protein binds heme *in vivo*. PduO forms inclusion bodies, so its isolation required high concentrations of urea with the loss of any coordinated heme. We could, however, incubate this PduO with heme B and look for the effect in UV/Vis spectroscopy. The PduO-heme spectrum shows the Soret band at 411 nm, as well as two additional bands at 528 and 560 nm, indicating the formation of PduO:heme *in vitro* (Figure S4B). Evidently, full-length PduO binds heme, but the C-terminal domain is responsible.

### Does PduOC bind cobalamin?

PduOC is related to HbpS from *Streptomyces*, but HbpS binds aquo-cobalamin (H_2_OCbl) as well as heme and iron (Wedderhoff et al., 2013; Ortiz de Orué Lucana et al., 2014). Iron-binding assays showed no evidence of iron binding (results not shown). To check for cobalamin binding, PduOC apoprotein was mixed with an excess of different cobalamins (AdoCbl, CNCbl, MeCbl, and H_2_OCbl) in the dark or during exposure to laboratory light at 37°C for 2 h. Mixtures were loaded onto a native PAA gel (Figure S5A). During electrophoresis, a pink band migrated in samples containing H_2_OCbl which were mixed in the dark and when lit. All Cbl protein-free species migrated together with the dye front. Pink bands were also seen with MeCbl and AdoCbl, but only in the samples incubated under laboratory lighting (Figure S5A, top). It is known that MeCbl and AdoCbl undergo photolysis in oxygenated solutions yielding H_2_OCbl (Pratt, 1972). The interpretation is that the pink band is a PduOC-H_2_OCbl complex. Over the time of the electrophoresis, the pink bands completely disappeared (Figure S5A, middle). This probably reflects weak binding. Page-Blue gel staining after electrophoresis showed the presence of PduOC proteins on the gel (Figure S5A, bottom). This gel shows that proteins in samples that interacted with H_2_OCbl appear to have a slightly higher molecular mass.

Binding of H_2_OCbl was also checked by UV/Vis spectroscopy. As a positive control we used the *Streptomyces* HbpS protein as this is known to bind H_2_OCbl (Ortiz de Orué Lucana et al., 2014). The HbpS sample showed the expected shift of the γ peak of H_2_OCbl from 352 to 358 nm (Figure S5B). With PduOC, there was an insignificant shift to 354 nm, suggesting a non-specific binding.

If PduOC binds H_2_OCbl, it does so with extremely weak affinity.

### Does PduOC influence the activity of the PduO catalytic domain *in vitro*?

The N-terminal domain of PduO is responsible for the protein's ATP:Cob(I)alamin adenosylcobalamin (ACA) transferase activity, but it is possible that the C-terminal domain has some effect on the catalysis. To investigate this, PduO and PduON were isolated from inclusion bodies using 4 M urea which was subsequently diluted, first to 3 M and then to 2 M, by dialysis. The refolding of PduO was followed by tryptophan fluorescence spectroscopy (Figure S6). In 4 M urea the maximal relative fluorescence intensity (133.1) was recorded at 351 nm. In 3 M a blue shift to 347 nm occurred with almost the same maximum intensity of fluorescence. However, in 2 M urea an additional blue shift to 336.6 nm occurred with considerably enhanced fluorescence intensity (171.2). The blue shifts and increased fluorescence indicate protein refolding. Similar spectra were recorded for PduON and PduOC apoprotein during their refolding (not shown). Subsequently, ACA activity using cob(I)alamin was assayed under anaerobic conditions for five protein combinations: full length PduO; PduON; PduOC holoprotein; PduON + PduOC apoprotein; PduON + PduOC holoprotein. In each case, 15 μg of each protein type was used. Mixtures with PduOC were incubated for 1 h at 37°C before the start of the assays. Activity was followed by the disappearance of cob(I)alamin (at 388 nm) and the appearance of AdoCbl (525 nm) over 20 min measured at 5 min intervals. In the sample containing PduOC holoprotein alone, no disappearance of cob(I)alamin was recorded (not shown). The spectra of the other four samples were similar (Figure S7). We then used Δε_388_ = 24.9 cm^−1^mM^−1^ (Johnson et al., 2001; Leal et al., 2004) to measure the AdoCbl concentration after 10 min, before the reaction ran to completion. For the full length and N-terminal domain, this was 15 ± 1 and 15 ± 1 μM, respectively. For the N-terminal domain with apo- or holo-protein, this was 15 ± 1 and 16 ± 1 μM, respectively. Under these conditions, the C-terminal domain had no measurable effect on the ACA activity of PduO. These data are consistent with previous results suggesting that only the N-terminal is necessary for PduO ACA activity (Johnson et al., 2004). We would, however, emphasize that for ACA assays, we and others used the strong reducing agent titanium(III) citrate to obtain cob(I)alamin. In cells, there are certainly other compounds responsible for this and other cobalamin tranformations (Chowdhury et al., 2014).

### Crystal structure of PduOC and heme coordination

The PduOC:heme complex crystallized in the P2_1_ space group with 2 octamers in the asymmetric unit (PDB acquisition code 5CX7^1^). All 16 independent protein monomers had almost identical structures. Experimental conditions, data collection parameters and processing/refinement statistics from the last refinement cycle are outlined in Table 1.

**Figure 5:**
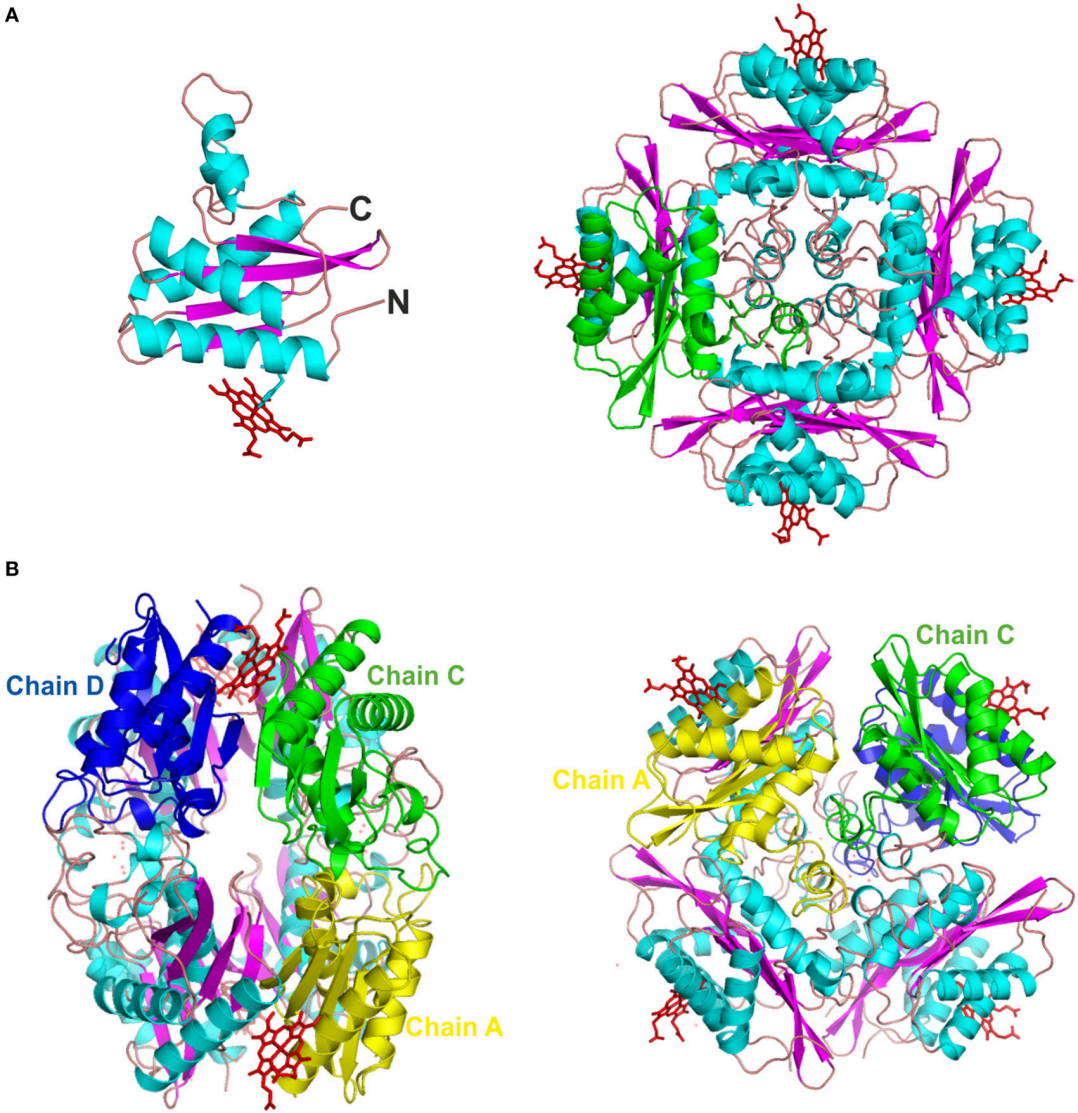
**The 3D crystal structure of PduOC:heme**. **(A**, left) An illustration showing the PduOC monomer structure. The heme is indicated at the side chain of His18 that is shown in stick representation. α-helices are shown in turquoise and β-strands in purple. **(A**, right) Octameric PduOC:heme assembly viewing as viewed down the main C_4_ axis. A single PduOC monomer is highlighted in green. **(B)** Contacts between the three non-redundant monomeric PduOC molecules (chains A, C and D) within the octameric assembly as labeled.

The PduOC monomer has 147 residues, but the nine N-terminal residues were not modeled as this was not justified by the electron density. Each monomer has a three-layer, α-β-α folding that resembles the profilin fold (Murzin et al., 1995). More specifically, it is composed of the sequence α-β(2)-α(3)-β(2)-α (GlcG-like superfamily). This is a basic unit of four antiparallel β-strands that separate a couple of antiparallel α-helices (one at the N-terminal and one at the C-terminal) from a sequence of three α-helices (Figure 5A, left). In the case of these three helices, two are considerably shorter than the third, which interacts with the β-sheet. The overall quaternary arrangement is very similar to OrfY (PDB: 2A2L), and the heme-binding protein HbpS (PDB: 3FPV), both of which also crystallize as octamers (Ortiz de Orué Lucana et al., 2009).

The octamer structure buries a large fraction of the monomers, leaving the two antiparallel α-helices exposed (Figure 5A, right), but hiding the other three helices from the solvent. Quantifying this, more than 57% of the total surface area is hidden (Krissinel and Henrick, 2007), but this is not due to hydrophobic burial. Across one interface, the β-sheets from monomers are hydrogen bonded, so as to form a large, regular 8-stranded sheet spanning pairs of dimers. There are also numerous interactions involving polar and charged residues. One can also make some guesses as to lower-order oligomeric states. Due to the internal symmetry of the octamer, only two non-redundant dimer possibilities exist: CD and AC (Figure 5B). At 768 Å^2^, the AC contact area is 42% larger than the CD contact area, but the CD pair contains the extended β-sheet and heme-binding site. The octamer structure is dependent on heme (discussed below), so it seems likely that the CD dimers are most stable and brought together, in the presence of heme, by AC contacts and the possibility of burying another 240 Å^2^ of surface area.

**Figure 6:**
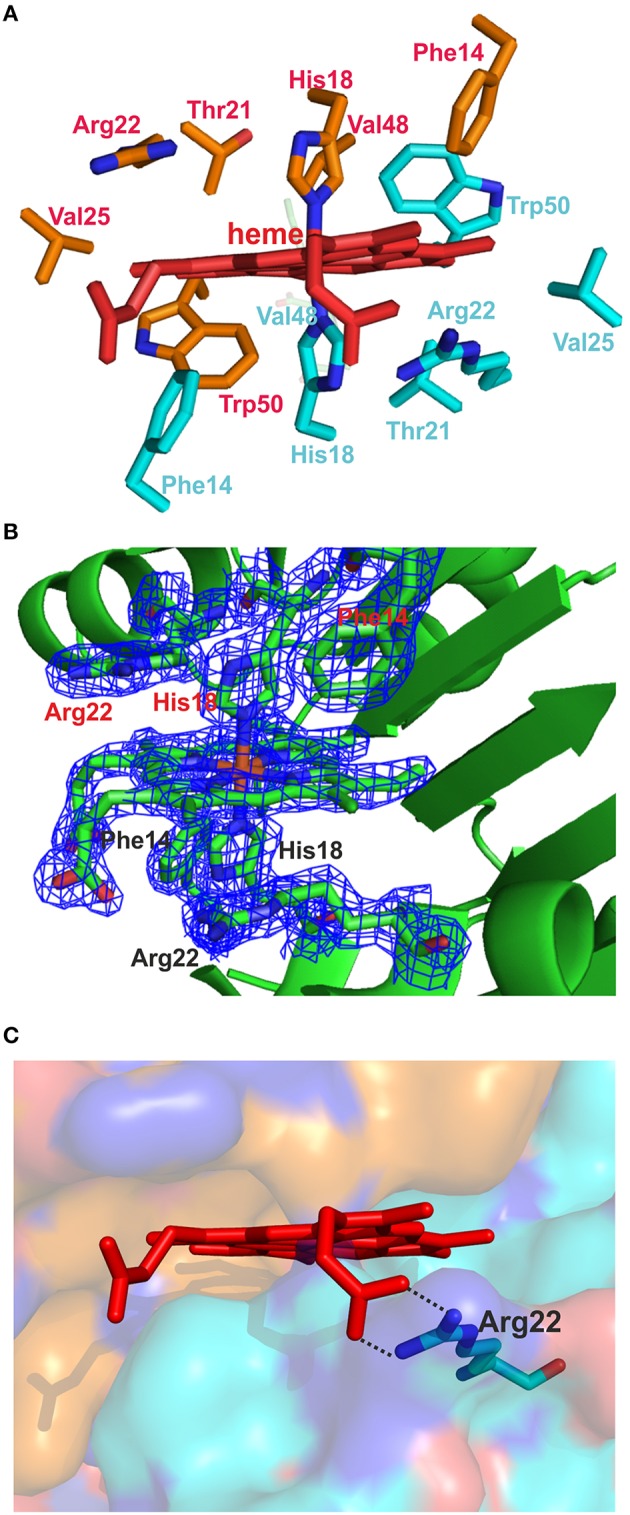
**The heme-binding pocket. (A)** The amino acids from two adjacent subunits forming the heme-binding pocket are indicated. **(B)** The heme, the two His18 iron-binding residues, and the residues Arg22 and Phe14 from each chain are shown as sticks, along with their associated electron density maps (2*F*_*o*_ − *F*_*c*_) contoured at 1.5 σ. **(C)** The surface of one heme-binding pocket and the heme are shown. The backbone of one monomer is colored with turquoise and that of the adjacent monomer with bronze. The interactions between Arg22 and the heme propionate are indicated with black dotted lines.

Each octamer binds four heme molecules (Figures 5A,B) with each heme sitting in a pocket formed by the extended β-strand and an α-helix from each of two dimers. Given that this interface is held together by hydrogen bonds (in the sheet) and since it is filled by a heme, it could not be called a hydrophobic interface. The eight heme groups in the asymmetric unit are highly exposed, with solvent accessible areas from 251 to 302 Å^2^. The heme iron is hexa-coordinated with *bis*-histidyl (*bis*-His) ligation by two histidines (H18) from adjacent monomers (Figures 6A–C). The symmetry is such that pairs of corresponding residues from adjacent chains form the pocket (Phe14, His18, Thr21, Arg22, Val25, Val48, and Trp50; Figures 6A,B). There are slight differences in the orientation of the eight heme molecules in the asymmetric unit, with small rotations of the porphyrin ring, but one should be cautious with the interpretation, given the poor electron density of some of the external heme carboxylic groups within the octamer. Presumably this reflects heme mobility and disorder, and crystal twinning. Figure 6B shows one heme-binding pocket in which the guanidinium moiety of Arg22 interacts with one propionate from the heme ring via a double salt bridge (2.4 and 2.8 Å in Figure 6C) although this was not evident for all the heme pockets.

**Figure 7:**
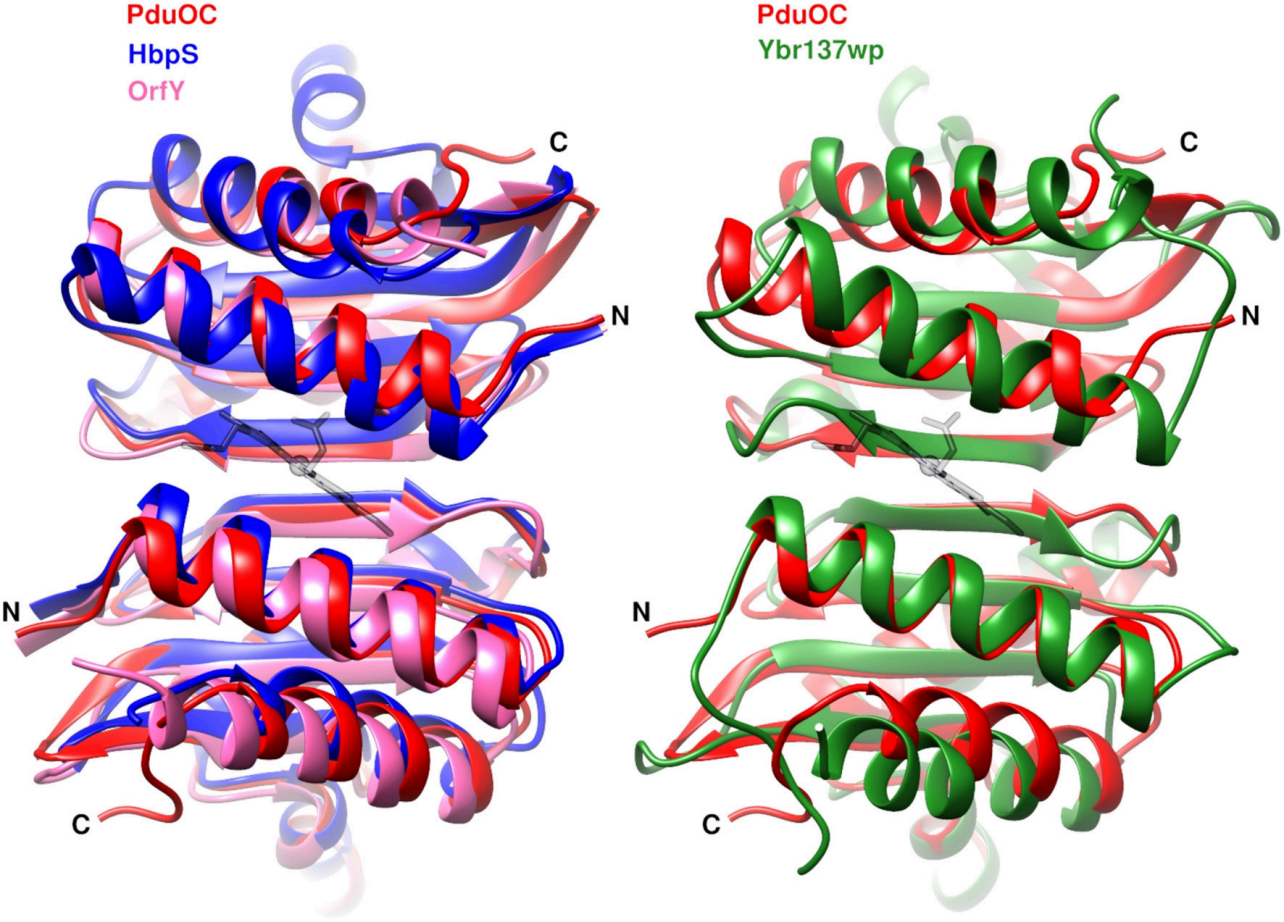
**Dimer similarities from bacterial octamers (left) and the eukaryotic decamer (right)**. Label colors correspond to protein colors. The heme (gray) coordinates come from 5CX7. PDB codes PduOC: 5CX7, HbpS: 3FPV; OrfY: 2A2L; Ybr137wp: 4CLC.

A search for structural homologs gives the expected sequence homologs (OrfY and HbpS) as well as a fungal protein, Ybr137wp (Figure 7). In each case, the binding pocket formed by a dimer is conserved. This is even the case for the fungal protein structure in which the dimer is part of a decamer and there is absolutely no evidence of heme binding or any involvement in redox chemistry (Yeh et al., 2014). Although the shape of the pocket is conserved, the sidechains are different. From the structural alignment, the heme-coordinating histidine is replaced by a threonine or glutamine in the bacterial proteins and by phenylalanine in the fungal protein (Table S4).

### PduOC is an octamer in solution

**Figure 8:**
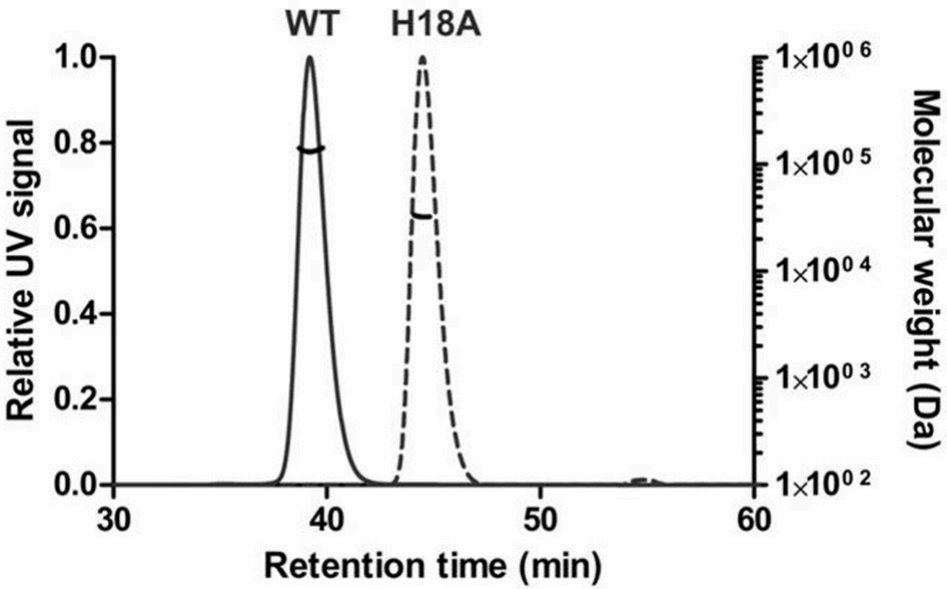
**Oligomeric state of PduOC proteins in solution**. The molecular weights of the wild-type (WT) and the PduOC-H18A (H18A) mutant protein were analyzed by SLS after gel filtration chromatography.

SLS, coupled to size exclusion chromatography was used to check the oligomerization state of PduOC:heme in solution. It eluted as a single symmetric peak with a molecular mass of 134,700 ± 800 Da, close to the value of 125,391 Da expected for an octamer with 4 bound heme molecules (Figure 8). We assume that the difference of ~9000 Da between the expected and observed molecular mass of PduOC:heme is likely due to the presence of heme. The molecular mass of the octamer can be contrasted with the H18A mutant which does not bind heme and had a molecular mass of 32,500 ± 100 Da estimated from SLS (Figure 8). Furthermore, gel filtration of the PduOC apoprotein showed a large dimer peak (not shown). Taken together, it appears that PduO:heme forms octamers which are very stable in solution. The exchange of His18 by Ala abolishes heme binding and this shifts the equilibrium from octamers to dimers.

### Does the heme-binding residue in PduO influence the catabolism of 1,2-propanediol?

From the crystal structure, one knows that His18 is the iron-coordinating residue. From the biochemical and mutation data, one knows that the residue is essential for heme-binding and octameric assembly. This raises the question as to the role of His18 in the full-length protein *in vivo*. We constructed the plasmid pWKS30RO-H18A, carrying the complete *pduO* gene with the codon for His18 (nomenclature as in PduOC) replaced by one for Ala. This was used to transform *S. enterica* Δ*pduO* Δ*cobA*. The growth of this transformant (+PduO-H18A) was compared to that of *S. enterica* Δ*pduO* Δ*cobA* transformed with pWKS30RO (+PduO), carrying the native *pduO* gene. After 36 h of cultivation at 37°C on MacConkey-1,2-propanediol-CNCbl indicator agar, colonies of the +PduO strain showed a red color in the indicator medium. +PduO-H18A colonies were also red-colored, but with considerably less intensity. After 50 h of incubation, the red color intensity was enhanced in both strains (Figure S8A). We subsequently compared the growth of the strains in PCN minimal medium containing 1,2-propanediol and CNCbl. *S. enterica* Δ*pduO* Δ*cobA* and *S. enterica* Δ*pduO* Δ*cobA* pWKS30RON (+PduON) were used as a control. As expected, the Δ*pduO* Δ*cobA* mutant had a very low growth rate compared to the +PduO strain (Figure S8B). The growth of the +PduO-H18A strain showed a comparable growth behavior as +PduO, but its final optical density (OD) of 3.5 was slightly lower than that of +PduO (4.8). Interestingly, the final OD of +PduO-H18A lies in between of +PduO and +PduON strains, the second with a final OD of 2.9 (Figure S8B).

The growth assays suggest that the mutation of the heme-binding histidine residue in PduO led to less effective catabolism of 1,2-propanediol.

## Discussion

There are more than 3600 entries in the protein databank with some form of heme (Berman et al., 2000) so it is a pleasure to find a new binding mode. Because of its strong similarity to OrfY, PduOC would be called an example of the profilin fold (Murzin et al., 1995). The sensor proteins, FixL and DosP, are also members of this fold family and also bind heme (Gong et al., 2000; Miyatake et al., 2000; Kurokawa et al., 2004; Key and Moffat, 2005). What is completely different is the binding mode. The sensor proteins bind heme within a monomer, between an α-helix and β-sheet and do not have *bis*-His heme ligands. The pocket in PduOC is in a completely distinct part of the structure, between α-helices from different monomers and sitting on top of a β-sheet running across dimers. Considering the complete two-domain protein, the structure is even more interesting. While multi-heme proteins are well known, a protein which binds a cobalt-tetrapyrrole (in its N-terminal domain) and an iron-tetrapyrrole (C-terminal domain) is a real rarity.

This leads to the question of what the PduO C-terminal domain does *in vivo*. There was no evidence from the enzyme assays that it directly affected the whole protein's cobalamin adenosyltransferase activity, although one cannot rule out the necessity for some other ligand, regulator or influence to show an effect. At the same time, the deletion mutant showed that the C-terminal domain and its heme binding are necessary for effective *in vivo* catabolism of 1,2-propanediol. The sequence conservation calculation suggests that there are sites in the C-terminal domain experiencing just as much evolutionary pressure as in the N-terminal domain. The results are not contradictory. It means that PduOC has some accessory role, such as providing or transforming a co-factor. Heme proteins involved in catalysis or oxygen transport usually have just one amino acid as an axial iron ligand. Their iron is either penta-coordinated or has water at the sixth coordination site in the resting state. In contrast, heme proteins that are involved in electron transfer reactions usually have both axial ligands donated by amino acid residues (Zoppellaro et al., 2009).

The *bis*-His heme coordination in PduOC is consistent with a possible role in electron transfer. First, the heme groups within octameric PduOC are highly solvent-exposed. This has been suggested to be a characteristic of heme proteins (including cytochrome *c*_3_ and cellobiose dehydrogenase) involved in electron transfer (Stellwagen, 1978; Czjzek et al., 1994; Hallberg et al., 2000; Smith et al., 2010). Second, the UV/Vis spectroscopy showed that the heme in PduOC is redox-active.

Looking at the >20 genes of the 1,2-propanediol usage (*pdu*) operon, most gene functions are involved in cobalamin manipulation or a proposed role in forming the micro-compartment shell, rather than metabolite transformation. Amongst the proteins on the operon is a cobalamin reductase, known as PduS, which is essential for cobalamin recycling and exploiting cobalamin derivatives which have been taken up. PduS uses flavin mononucleotide (FMN) and NADH co-factors and has motifs typical of iron-sulfur-cluster binding (Cheng and Bobik, 2010). PduS catalyzes the reduction of cob(III)alamin and cob(II)alamin *in vitro*. It was suggested that only the cob(II)alamin reductase is relevant *in vivo*, while cob(III)alamin is reduced to cob(II)alamin *via* an undescribed chemical reduction in the cytoplasm or within the micro-compartment (Sampson et al., 2005; Chowdhury et al., 2014). Interestingly, cobalamin reductases are believed to be electron transfer proteins, not enzymes (Mera and Escalante-Semerena, 2010). Given that the ATP:Cob(I)alamin transferase PduO physically interacts with PduS (Cheng and Bobik, 2010), we suggest that the C-terminal domain of PduO might play an important role in the set of cobalamin transformations. Further studies are necessary to characterize the redox reactions in which the heme groups at the surface of PduOC, cobalamin and/or other co-factors might be involved.

The most surprising feature of PduOC:heme is the structural conservation of the heme-binding pocket in proteins from bacteria to eukaryotes, across different multimeric assemblies and spanning different functions (Figure 7). The fungal protein Ybr137wp is a decamer, rather than an octamer, but it has the same dimer unit with the extended β-sheet and binding pocket. On the evolutionary time-scale, while the overall multimer has been reorganized, the dimer unit has been conserved. One might ask if tetrapyrrole binding in Ybr137wp has been missed. On current evidence this is not likely. In a structural alignment, the fungal protein residue corresponding to the iron-coordinating histidine is a phenylalanine and there is no evidence of involvement in redox chemistry (Yeh et al., 2014).

This conservation of structure leads to the final issue. To what extent do the properties of PduO apply to related proteins? If one looks at closely related homologs, the properties are very transferable. Amongst the 400 closest homologs to full-length PduO, the heme-coordinating histidines are completely conserved. This is highlighted for a small group of enteropathogenic bacteria (Figure S9). It is safe to assume that if *S. enterica* PduOC binds heme, the corresponding proteins from these bacteria do as well. More interesting are the slightly less closely related relatives. One can calculate the sequence entropy for 456 two-domain, full-length PduO homologs. This would correspond to the two-domain proteins in Figure 2 and is shown in Figure S1. Now His18 is often replaced by lysine and glutamate and arginine. There are few proteins (e.g., cytochrome c, quinol dehydrogenase, named NrfH) that use lysine as heme axial ligand (Rodrigues et al., 2006). In some other heme proteins (i.e., cytochrome P450 enzymes) glutamate and arginine were found to interact with the heme moiety, but not as axial ligands (Hasemann et al., 1995; Lebrun et al., 2002). Thus, it is likely that the PduO homologs either do not bind heme or have very unusual coordinating residues, or the structure adapts to allow interactions with neighboring residues. The story becomes even stranger. From the structural alignments, the corresponding residue in HbpS is clearly a threonine. This protein does bind heme, albeit with a dissociation constant four orders of magnitude weaker, but has resisted all attempts to persuade it co-crystallize with heme and it has been named a heme-degrading domain (Ortiz de Orue Lucana et al., 2009; Finn et al., 2014).

**Figure 9:**
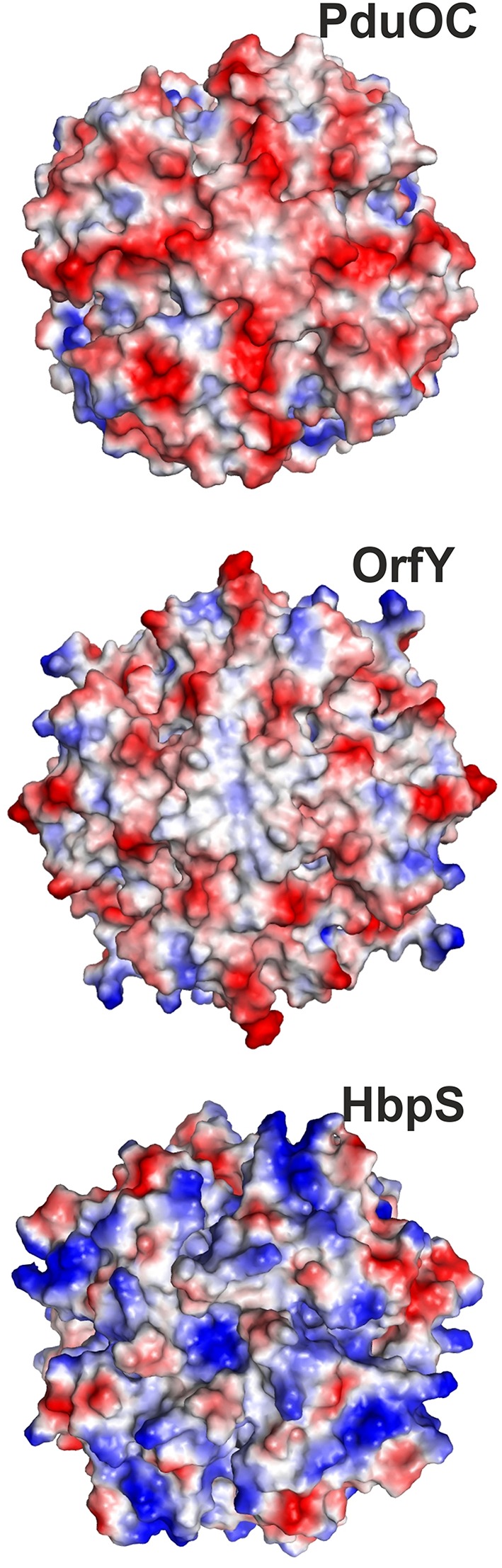
**Charges on surfaces of octamers of PduOC (top), OrfY (middle), and HbpS (bottom) with negative (red) and positive (blue)**.

Returning to the bacterial proteins, there is good reason to expect functional differences amongst the bacterial protein families. Firstly, there are more proteins than one might realize. The proteins in the tree in Figure 2 result from searching for C-terminal domain homologs. This means they include the closest (two-domain) proteins as well as C-terminal-domain homologs. Had one used the N-terminal domain, one would have seen the two-domain proteins and a set of N-terminal homologs. In some organisms, such as *K. pneumoniae*, one finds the two-domain protein, an N-terminal homolog and a C-terminal homolog. This could be redundancy, but is more likely to be a set of proteins with different specificities and roles. There are also differences in the biochemical properties. PduOC is intracellular and might even reside in a protein micro-compartment. In contrast, HbpS which seems to have diverged earliest (Figure 2), is a secreted protein. Finally, the octameric structures present very different surfaces to the outside solution. Figure 9 shows the octamers with their surfaces color-coded by charge. PduOC is negatively charged, OrfY less so and HbpS more positively charged. In the case of PduOC, the surface is even more different, since it must host the N-terminal domain. Obviously a crystal structure of the full-length protein would be most informative.

The work finishes with some questions that will eventually be answered. Given PduO's context in the *pdu* operon and its interaction with a cobalamin reductase (PduS), can one measure its influence or catalysis of some related step? Which role does the heme group play in these reactions? We have detected binding to heme B, but there is always the possibility that the protein's real role involves some related tetrapyrrole. Even when the function of both domains in PduO becomes clearer, it remains to be seen if this explains the roles of the single-domain homologs of the N- and C-terminal domains. Finally, the crystal structure of the full-length protein should be solved, but we would wager that it maintains the large β-sheet spanning two dimers and preserves the heme-binding site.

## Author contributions

All authors substantially contributed to the work reported. DO conceived and coordinated the study. DO, NH, MH, and AT designed experimental strategies. DO, NH, SG, MH, JK, TT, and AT performed experiments and analyzed data. DO and AT wrote the paper.

### Conflict of interest statement

The authors declare that the research was conducted in the absence of any commercial or financial relationships that could be construed as a potential conflict of interest.

## Footnotes

^1^The coordinates of the crystal structure of PduOC:heme have been deposited in the PDB, code 5CX7. The atomic coordinates and experimental data will be released immediately upon publication.

## Abbreviations

*bis*-His: *bis*-histidyl
Cbl: cobalamin
SLS: static light scattering
WT: wild type
PAA: poly-acrylamide
PDB: protein data bank.
