# Supplementary material for: The Crystal Structure of the C-Terminal Domain of the Salmonella enterica PduO Protein: An Old Fold with a New Heme-Binding Mode
Source: Front Microbiol. 2016 Jun 28;7:1010. doi: 10.3389/fmicb.2016.01010 (PMC4923194; doi:10.3389/fmicb.2016.01010)
Supplement: Supplementary file 1 [file Table1.PDF]

Table S1. List of *Salmonella* strains used in this study

Designation	Genotype/characteristics	Source/reference
Salmonella enterica serovar Typhimurium		
NCTC12023	wild type	lab stock
MvP2087	Δ*cobA*::*aph*, *cobA* deletion, kanamycin resistance	this study
MvP2091	Δ*cobA*::FRT, *cobA* deletion	this study
MvP2089	Δ*pduO*::*aph*, *pduO* deletion, kanamycin resistance	this study
MvP2093	Δ*pduO*::FRT, *pduO* deletion	this study
MvP2094	Δ*cobA*::*aph* Δ*pduO*::FRT, *cobA* deletion, kanamycin resistance, *pduO* deletion	this study

Table S2. List of plasmids

Designation	Genotype/characteristics	Source/reference
pKD13	Template plasmid for *aph*/FRT cassette	(Datsenko & Wanner, 2000)
pWRG730	Temperature inducible expression of *red*αβγ, ts ori	Lab stock
pCP20	FLP expression, ts ori	(Datsenko & Wanner, 2000)
pWSK30	Low-copy plasmid used to clone *pduO* for complementation studies in *Salmonella*	(Wang & Kushner, 1991)
pWSK30R	*pduA* promoter (P_{pduA}) in pWKS30	This work
pWSK30RO	pWSK30R containing the complete *pduO* gene downstream of P_{pduA}	This work
pWSK30RN	pWSK30R containing the DNA region encoding the N-terminal domain of PduO downstream of P_{pduA}	This work
pWSK30RC	pWSK30R containing the DNA region encoding the C-terminal domain of PduO downstream of P_{pduA}	This work
pWKS30RO-H18A	pWSK30RO encoding PduO-H18A	This work
pETM11	Expression vector	(Zou *et al*., 2008)
pETPduO	pETM11 encoding full PduO	This work
pETPduO-H18A	pETPduO encoding PduO-H18A	This work
pETPduON	pETM11 encoding N-terminal domain of PduO	This work
pETPduOC	pETM11 encoding C-terminal domain of PduO	This work

pETPduOC-H15A	pETPduOC containing *pduOC* H15A	This work
pETPduOC-H18A	pETPduOC containing *pduOC* H18A	This work
pETPduOC-H43A	pETPduOC containing *pduOC* H43A	This work
pETPduOC-H78A	pETPduOC containing *pduOC* H78A	This work
pETPduOC-H96A	pETPduOC containing *pduOC* H96A	This work
pETPduOC-H146A	pETPduOC containing *pduOC* H146A	This work

Table S3. List of primers

Designation	Sequence (5' → 3')
PRegForEco	GTTGAATTCGGAAAAATCGTGTTGCG
PRegRevNcoHind	CGCAAGCTTGACCCATGGAAAGACCTCGCATGGAGTGTTC
POForNco	CACCATGGCGATTTATACCCGAAC
POrevHin	GTCAAGCTTTCATTGATGAGTTCCCACG
PNRevHin	GTAAGCTTTCAGCTGTGGGGCGGCTG
PCForNco	GTTCCATGGCCAAGGAGACGACGCCAGTGG
PForNcoH15A	CGCCATGGCCAAGGAGACGACGCCAGTGGCCCTCTCATTCGCCGATCTGCACCAG
PM11Rev	GGGCTTTGTTAGCAGCCGGATCTCAG
PM11For	GTGAGCGGATAACAATTCCCCTCTAG
PRevPstH18A	GCCTGCAGTTGCTGCGCGCGTTCAACGGCGGCGCGGGTGAGCTGGGCCAGATCGTGGAATG
PForPstH43A	CACTGCAGGTTCCGGTAGTCGTCAGCATCGTTGACGCGGCCGGCACGGAAAC
PforStuH78A	GAAGGCCTGGACCGCAGTGGCGATGAAAACGGCGACCGCCGAGCTGAGCGATG
PRevHinH146A	GCAAGCTTTCATTGAGCAGTTCCCACG
PForH96A	GGAAAGTGCCTTACAGGGAAAAGTG
PRevH96A	GTAAGGCACTTTCCAGACCGTAAAG
pduO-Del13-For	CGGCATTGTAGATACGCTTTCGTGTTAAGGGGCGGTTATGATTCCGGGGATCCGTCGACC
pduO-Del13-Rev	TCGAGTTCAGAAGTATTCATTGATGAGTTCCCACGTTAATTGTAGGCTGGAGCTGCTTCG
btuR-Del13-For	AACCGGGCCGTAAACCAGGAATCGCCCAATGAGTGATGAAATTCCGGGGATCCGTCGACC
btuR-Del13-Rev	CCGGATGGCGGCGCAAACGCCTTAATCCGGCCTACGGTTATGTAGGCTGGAGCTGCTTCG
k1-RedDel	CAGTCATAGCCGAATAGCCT
pduO-DelCheck-For	GATGAAGTGGCCGTGGACTC

pduO-DelCheck-Rev	TAGCGGGCACTGCTGATAAC
btuR-DelCheck-For	TTCCGACGGAAGATCCACAG
btuR-DelCheck-Rev	TCGTCGTATGAAAGCGAACC

Table S4. Residues corresponding to His-18 from PduOC from structural alignments with three flanking residues are shown on either side. Numbering corresponds to the PduOC structure.

name	PDB code	sequence						
		15	16	17	18	19	20	21
PduOC	5CX7	**H**	**D**	**L**	**H**	**Q**	**L**	**T**
HbpS	3FPV	**E**	**A**	**A**	**T**	**K**	**A**	**A**
OrfY	2A2L	**A**	**A**	**A**	**Q**	**Q**	**M**	**A**
Ybr137wp	4CLC	**Q**	**D**	**A**	**F**	**D**	**L**	**G**
