# Supplementary material for: The Crystal Structure of the C-Terminal Domain of the Salmonella enterica PduO Protein: An Old Fold with a New Heme-Binding Mode
Source: Front Microbiol. 2016 Jun 28;7:1010. doi: 10.3389/fmicb.2016.01010 (PMC4923194; doi:10.3389/fmicb.2016.01010)
Supplement: Supplementary file 2 [file Image1.PDF]

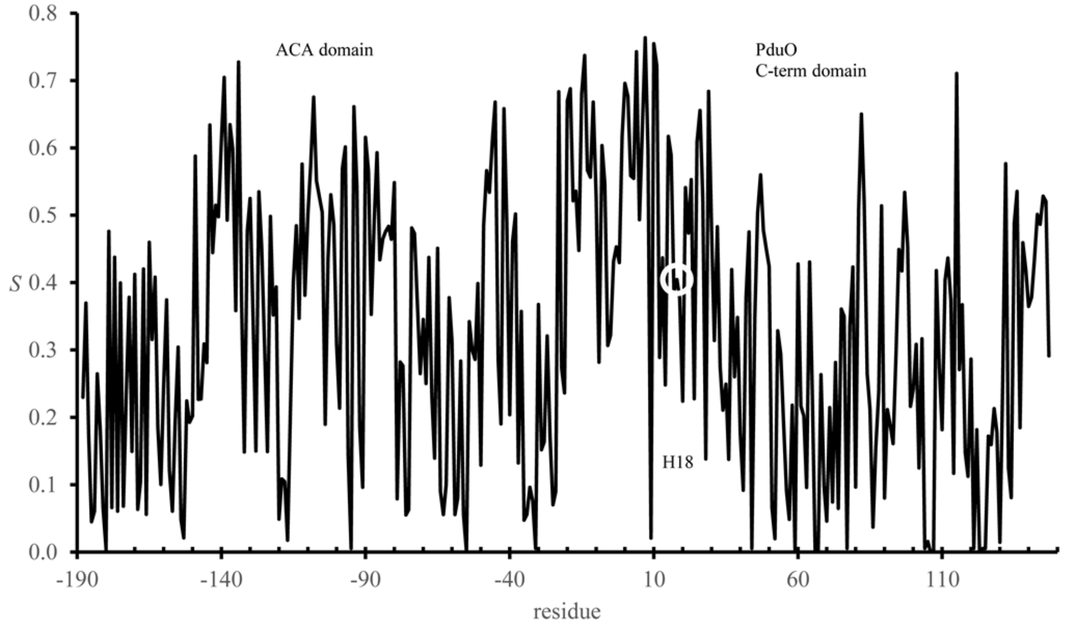

Figure S1. Sequence variability amongst 456 homologues of PduO. *S* is sequence entropy (eq. 1 in main text) with the scale ranging from 0 (conserved) to 1 (random amino acids). The white circle marks His18.
