# Supplementary material for: The Crystal Structure of the C-Terminal Domain of the Salmonella enterica PduO Protein: An Old Fold with a New Heme-Binding Mode
Source: Front Microbiol. 2016 Jun 28;7:1010. doi: 10.3389/fmicb.2016.01010 (PMC4923194; doi:10.3389/fmicb.2016.01010)
Supplement: Supplementary file 3 [file Image2.PDF]

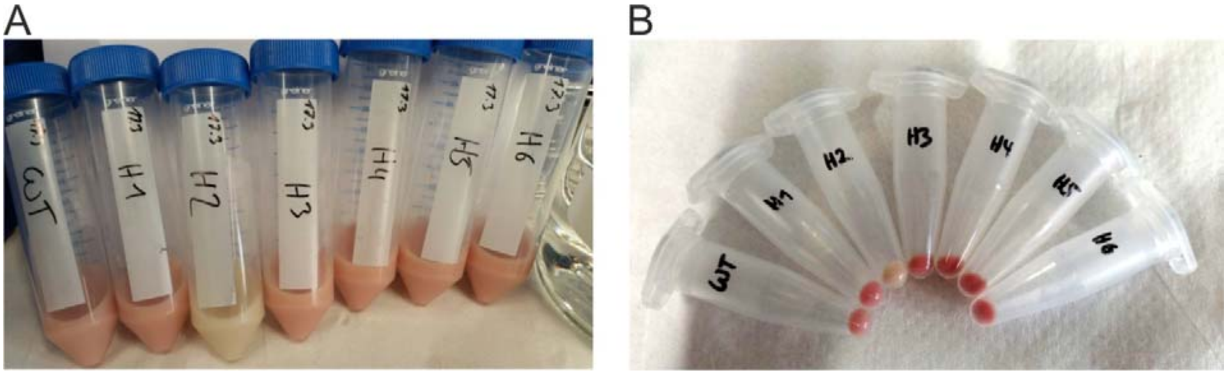

Figure S2. *E. coli* cultures producing PduOC wild-type (WT), PduOC-H15A (H1), PduOC-H18A (H2), PduOC-H43A (H3), PduOC-H78A (H4), PduOC-H96A (H5) and PduOC-H146A (H6), respectively, are shown prior (A) and after centrifugation (B).
