# Supplementary material for: The Crystal Structure of the C-Terminal Domain of the Salmonella enterica PduO Protein: An Old Fold with a New Heme-Binding Mode
Source: Front Microbiol. 2016 Jun 28;7:1010. doi: 10.3389/fmicb.2016.01010 (PMC4923194; doi:10.3389/fmicb.2016.01010)
Supplement: Supplementary file 4 [file Image3.PDF]

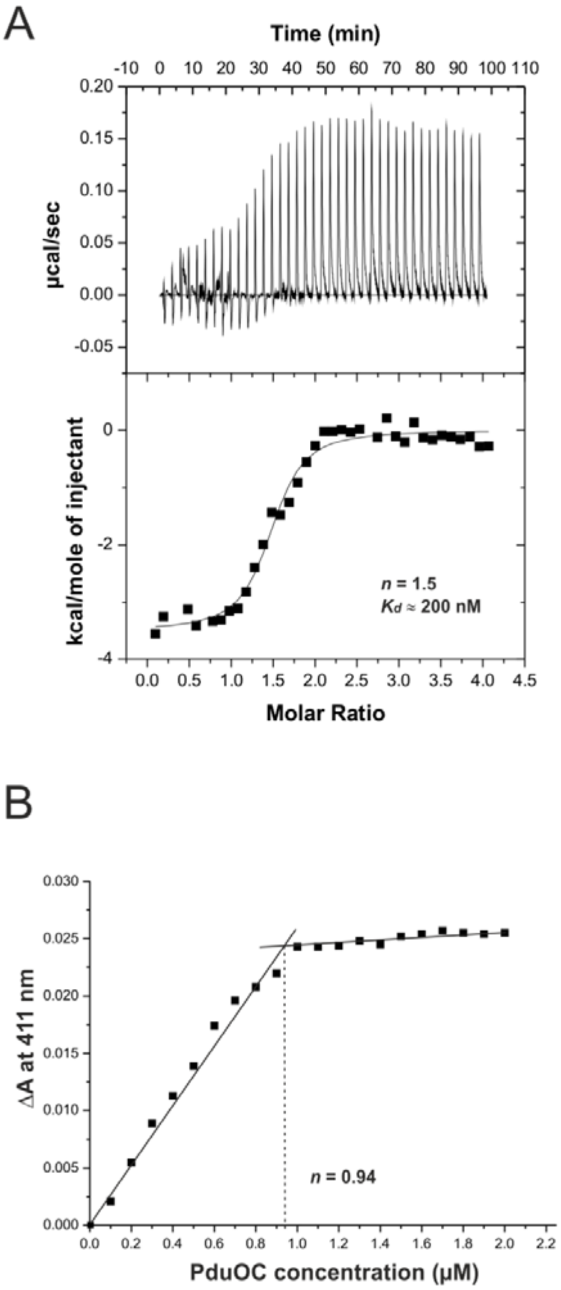

Figure S3. Heme binding by PduOC. (A) Binding isotherm for PduOC apoprotein titrated into heme. The apoprotein (200 µM) was injected stepwise (40x 7 µl) into a cell containing heme (10 µM) at 25 °C. Upper panel: Heat evolved upon injection of the apoprotein into heme as a function of injection order. Lower panel: Integrated heats of reaction plotted against the molar ratio of PduOC apoprotein to heme concentration. The calculated stoichiometry value *n* as well as the dissociation constant K_d are indicated. (B) Heme (0.5 µM) was incubated with increasing (0-2 µM, at 0.1 µM increments) concentrations of PduOC apoprotein and UV/Vis spectra were recorded. The plot shows the difference absorbance (ΔA) at 411 nm versus protein concentration. The dotted line indicates the apparent point of saturation. The deduced stoichiometry value *n* is indicated.
