# Supplementary material for: The Crystal Structure of the C-Terminal Domain of the Salmonella enterica PduO Protein: An Old Fold with a New Heme-Binding Mode
Source: Front Microbiol. 2016 Jun 28;7:1010. doi: 10.3389/fmicb.2016.01010 (PMC4923194; doi:10.3389/fmicb.2016.01010)
Supplement: Supplementary file 5 [file Image4.PDF]

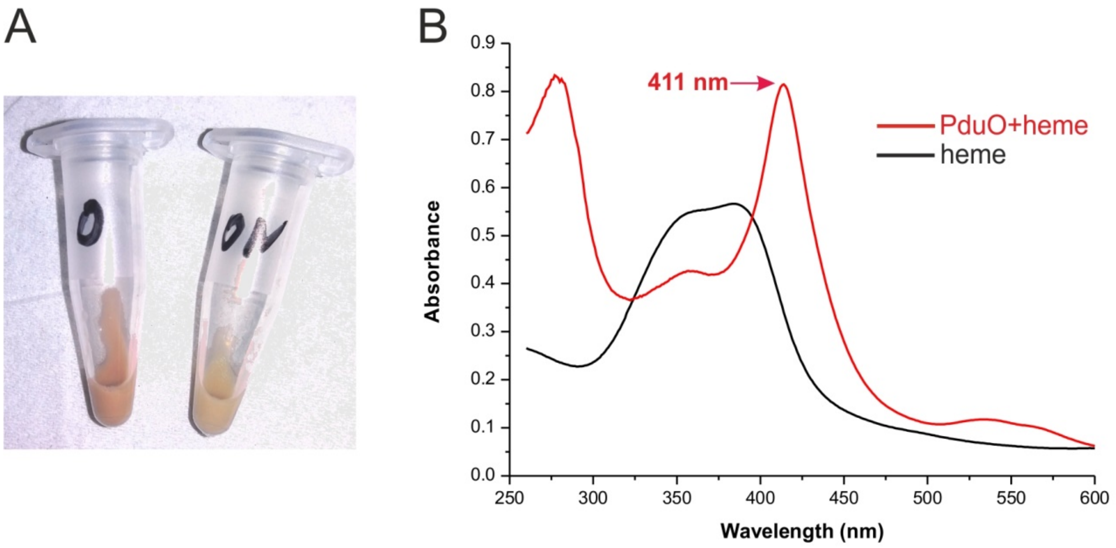

Figure S4. Heme binding by PduO. (A) Pellets of IPTG-induced *E. coli* cells producing either the two-domain PduO (O) or its N-terminal part (ON) are shown. (B) PduO protein (20 μM) was incubated with heme (10 μM) for 10 min at 25 ° C, and subsequently analysed by UV/Vis spectroscopy. Spectra of free heme (black) and of PduO+heme (red) are shown. The Soret band at 411 nm is indicated by an arrow.
