# Supplementary material for: The Crystal Structure of the C-Terminal Domain of the Salmonella enterica PduO Protein: An Old Fold with a New Heme-Binding Mode
Source: Front Microbiol. 2016 Jun 28;7:1010. doi: 10.3389/fmicb.2016.01010 (PMC4923194; doi:10.3389/fmicb.2016.01010)
Supplement: Supplementary file 6 [file Image5.PDF]

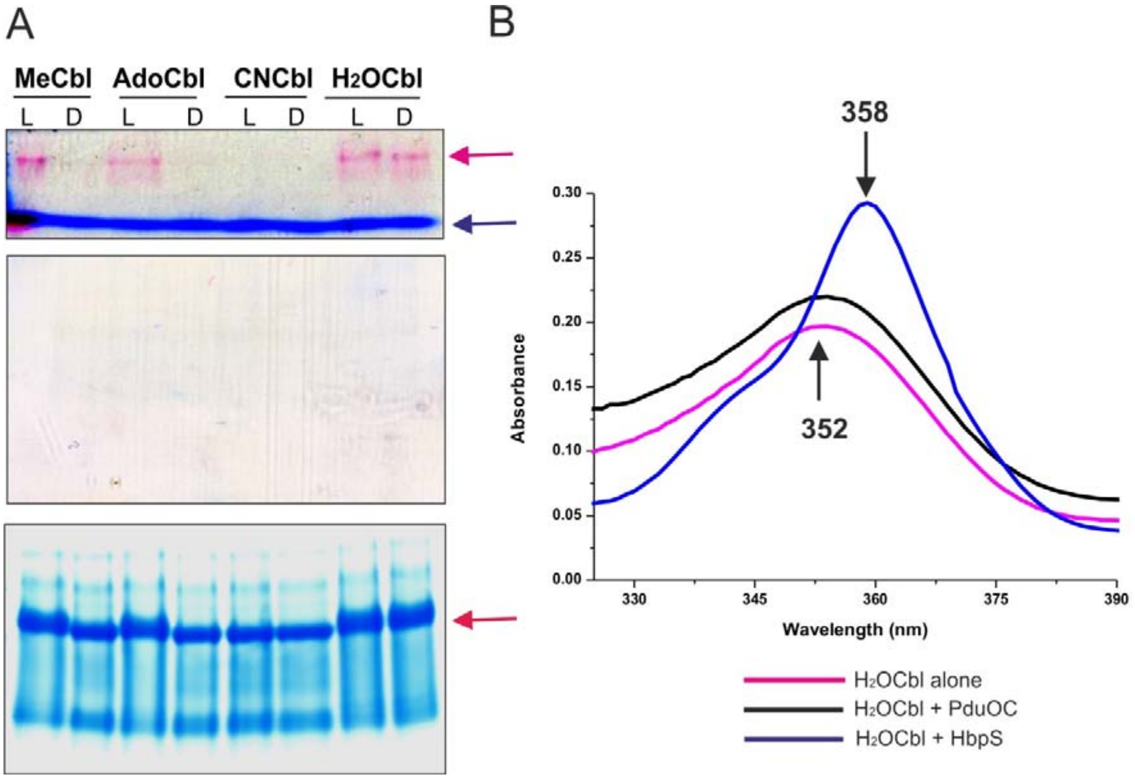

Figure S5. Cobalamin-binding assays. (A) PduOC was incubated with either aquo-cobalamin (H_2OCbl) or methylcobalamin (MeCbl) or adenosylcobalamin (AdoCbl) or cyanocobalamin (CNCbl) under exposure to ambient light (lanes L) or in the dark (lanes D). Reactions were loaded on to a native PAA gel that was photographed after ~30 min of electrophoresis (top). The pink arrow indicates the migrating PduOC-Cbl complex, whereas the blue one the dye front. After running, the gel was scanned (middle) and subsequently stained with PageBlue (bottom). The red arrow indicates the dominant form of PduOC on the native PAA gel. (B) Identical amounts (20 µM) of PduOC and the *Streptomyces* HbpS (control) were incubated with 10 µM of H_2OCbl as indicated under Experimental procedures. Spectra (in the range 325 to 380 nm) of H_2OCbl alone (pink spectrum) or of the samples additionally containing either PduOC (black spectrum) or HbpS (blue spectrum) are shown. The absorbance maxima at 352 nm and 358 nm are indicated by arrows.
