# Supplementary material for: The Crystal Structure of the C-Terminal Domain of the Salmonella enterica PduO Protein: An Old Fold with a New Heme-Binding Mode
Source: Front Microbiol. 2016 Jun 28;7:1010. doi: 10.3389/fmicb.2016.01010 (PMC4923194; doi:10.3389/fmicb.2016.01010)
Supplement: Supplementary file 7 [file Image6.PDF]

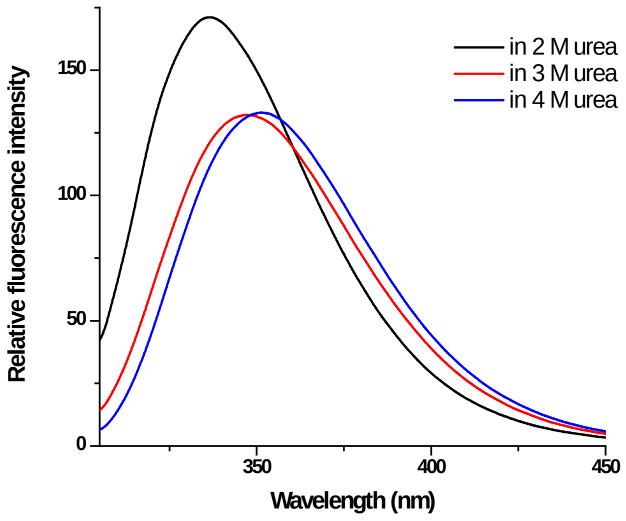

Figure S6. Tryptophan fluorescence of PduO. The fluorescence spectra of Histag-PduO (20 µM) in solution W containing either 4 M (blue) or 3 M (red) or 2 M (black) urea are shown.
