# Supplementary material for: The Crystal Structure of the C-Terminal Domain of the Salmonella enterica PduO Protein: An Old Fold with a New Heme-Binding Mode
Source: Front Microbiol. 2016 Jun 28;7:1010. doi: 10.3389/fmicb.2016.01010 (PMC4923194; doi:10.3389/fmicb.2016.01010)
Supplement: Supplementary file 8 [file Image7.PDF]

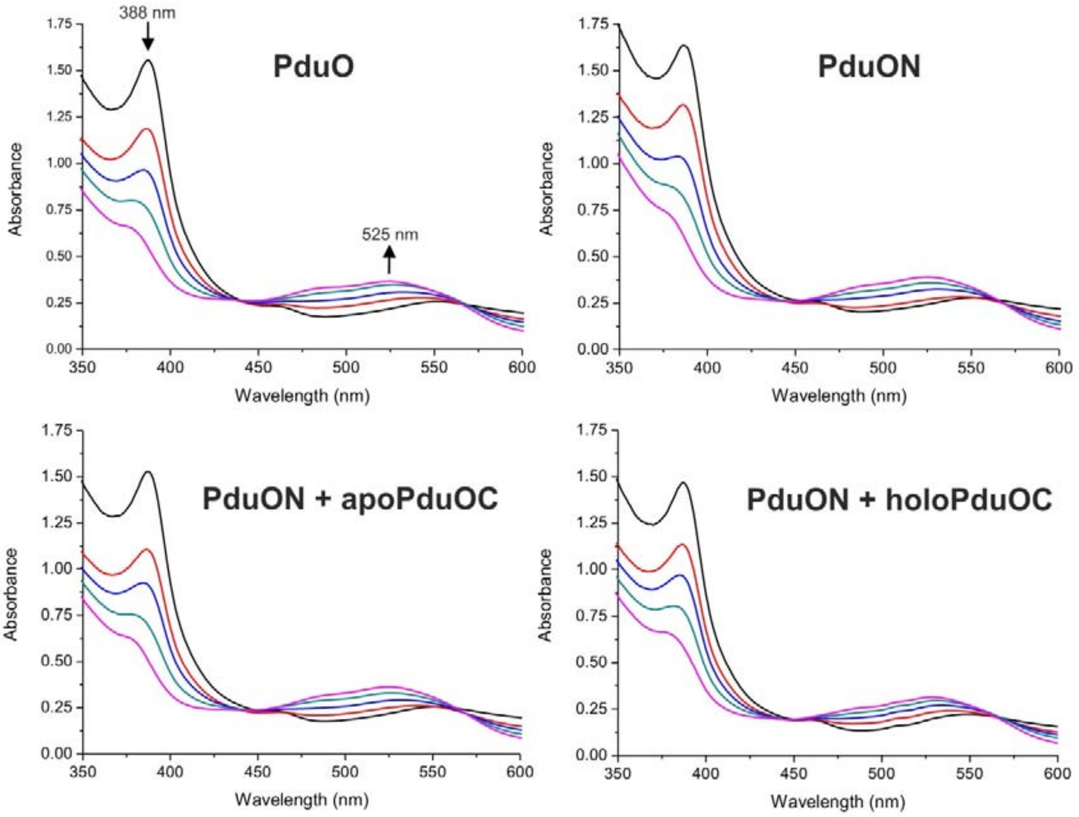

Figure S7. Analysis of AdoCbl synthesis *in vitro*. Absorption spectra (black, 0 min; red, 5 min; blue, 10 min, green, 15 min; pink, 20 min) of ACA assay mixtures containing the indicated protein(s) were recorded. An example of the decrease of cob(I)alamin at 388 nm and the appearance of AdoCbl at 525 nm are indicated by an arrow in the PduO sample.
