# Supplementary material for: The Crystal Structure of the C-Terminal Domain of the Salmonella enterica PduO Protein: An Old Fold with a New Heme-Binding Mode
Source: Front Microbiol. 2016 Jun 28;7:1010. doi: 10.3389/fmicb.2016.01010 (PMC4923194; doi:10.3389/fmicb.2016.01010)
Supplement: Supplementary file 9 [file Image8.PDF]

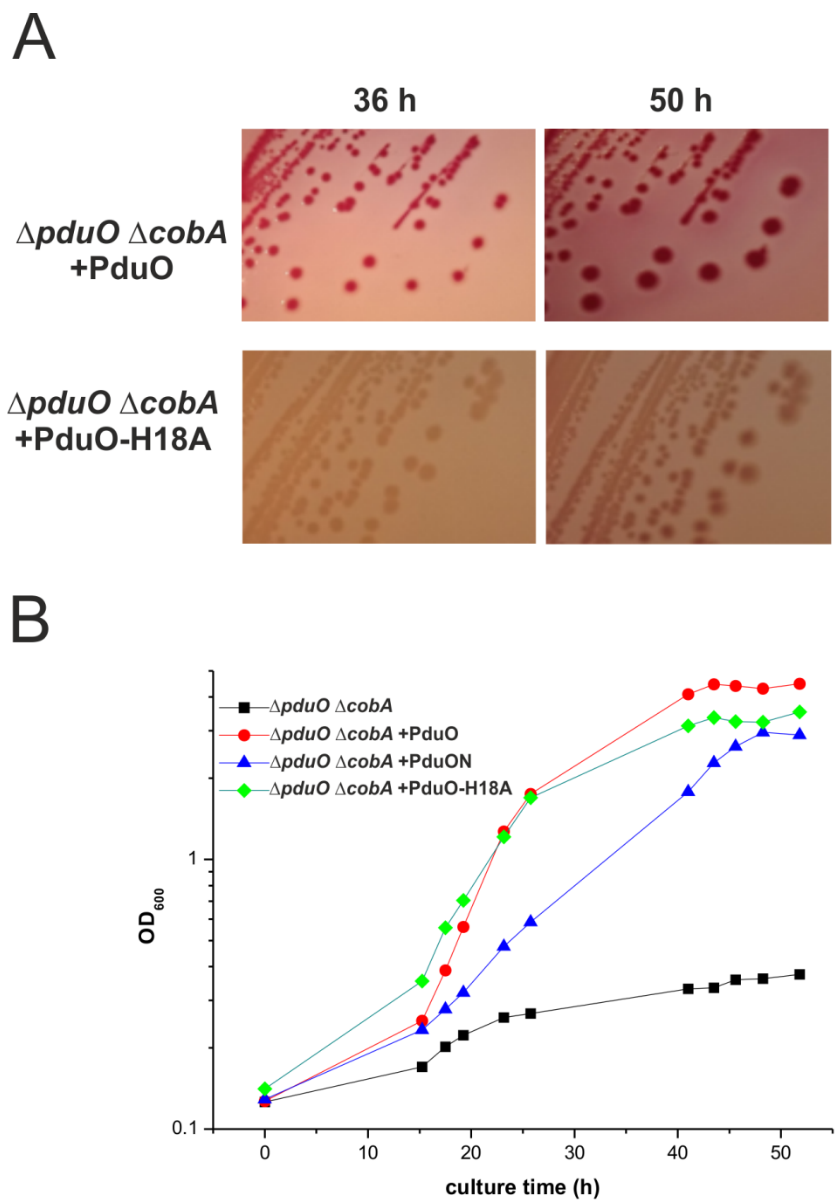

Figure S8. Growth of the *S. enterica* Δ*pduO* Δ*cobA* pWKS30RO (+PduO) and *S. enterica* Δ*pduO* Δ*cobA* pWKS30RO-H18A (+PduO-H18A), on MacConkey agar plates (A) or in minimal medium (B) both containing 1,2-propanediol and CNCbl is shown. The growth of *S. enterica* Δ*pduO* Δ*cobA* and *S. enterica* Δ*pduO* Δ*cobA* pWKS30RON (+PduON) strains is additionally shown in (B). Cultural growth (B) was determined by measuring optical density at 600 nm (OD_{600}) at various time points. Growth curves are representative for three independent assays with similar outcome.
