# Supplementary material for: The Crystal Structure of the C-Terminal Domain of the Salmonella enterica PduO Protein: An Old Fold with a New Heme-Binding Mode
Source: Front Microbiol. 2016 Jun 28;7:1010. doi: 10.3389/fmicb.2016.01010 (PMC4923194; doi:10.3389/fmicb.2016.01010)
Supplement: Supplementary file 10 [file Image9.PDF]

```
                    18
                    ↓
S_ente       GAMAKETTPVALSFHDLHQLTRAAVERAQQLQVPVVVSIVDAHGTETVTWRMPDALLVSSELAP
K_pneu          -KETTPVALSFLDLHQLTRAAVERAQQLQVPVVISIVDAHGTETVTWRMPDALLVSSELAP
K_oxyt          -KETTPVALSFHDLHQLTRAAVERAQQLKVPVVISIVDAHGTETVTWRMPDALLVSSELAP
C_freu          -KE-PAMSLSFQELHQLTRAAVTRAEELQVPVVISIVDANGTQTVTWRMPDALLVSSELAP
Y_fred          -AQPTIVGLGFPDVHQLVKLAVEAAMTLQIAVVVALADRHGNMIMTYRMPDTLLVSSELAP
Y_ente          -TQPASTGLGFSDVHQLVKLAVEAAMTLQIAVVVALTDRHGNMIMTYRMPDTLLVSSELAP
E_coli_1        -KERT-MPLTFQDLHQLIRSAAIRADELHIPVVISIVDANGTESVTWRMPDALLVSSELAP
E_coli_D        -KERT-MPLTFQDLHQLIRSAAMRADELHIPVVISIVDANGTESVTWRMPDALLVSSELAP
E_gall          -KAQA-GSLSFHELHQLIRQAIEHARQLQVPVVISIVDAHGTETVTWRMPDALLVSSELAP
                  :     * * ::*** : *   *  *:: **:::.* .*.  :*:****:*********

S_ente           KKAWTAVAMKTATHELSDVVQPGAALYGLESHLQGKVVTFGGGYALWRDGILIGGLGISG
K_pneu           KKAWTAVAMKTATHELSDVVQPGAALYGLESHLQGKVVTFGGGYALWRDGLLIGGLGISG
K_oxyt           KKAWTAVAMKTATHELSDAVQPGAALYGLETHLQGKVVTFGGGYALWRDGLLIGGLGISG
C_freu           KKAWTAVAMKTATHELTSAVQPGAALYGLESHMQGKVVTFGGGYALWREGLLLGGLGISG
Y_fred           KKAWTAVALKTATHQLNAAIQPGADLFQLEASTGGKVVSFGGGYPLWRGGKLVGGLGISG
Y_ente           KKAWTAVAMKTATHQLSAAIQPGADLFQLEASTGGKVVSFGGGYPLWRDGQLVGGLGISG
E_coli_1         KKAWTAVAMKTATHKLADAVQPGAPLYGLESHMQGKVVTFGGGFPLWRDGILLGGLGISG
E_coli_D         KKAWTAVAMKTATHKLADTVQPGAPLYGLESHMQGKVVTFGGGFPLWRDGKLLGGLGISG
E_gall           KKAWTAVAMKTATHELATTVQPGAALYGLESHLQGKVVTFGGGYPLWRDGQLIAGLGISG
                 ********:*****:*  .:**** *: **:   ****:****: *** * *:.******

S_ente           GSVEQDMDIAQTAIAAINVGTHQ
K_pneu           GSVEQDMDIAQTAIAAINVGTHQ
K_oxyt           GSVEQDMDIALAAIAAINVGTHQ
C_freu           GSVEQDMDIAETAIAAINVRTHQ
Y_fred           GSVAQDMHIAEAAISALHLRNE
Y_ente           GSVEQDMHIAEAAISALHLRNE
E_coli_1         GSVEQDMDIAQAAMAAINAGANQ
E_coli_D         GSVEQDMDIAQSAMAAINVGVNQ
E_gall           GSVEQDMAIAQAAMAAINVRTHQ
                 *** *** ** :*::*:.   .
```

Figure S9. Alignment of C-terminal domains of two-domain PduO proteins from different enteropathogenic bacteria. PduO C-terminal domains from *Klebsiella pneumoniae* (*K_pneu*; V3KG67), *Klebsiella oxytoca* (*K_oxyt*; A0A068HHK8), *Citrobacter freundii* (*C_freu*; A0A023VF82), *Yersinia frederiksenii* (*Y_fred*; A0A0B6FCC7), *Yersinia enterocolitica* (*Y_ente*; A0A0E1NHT0), *Escherichia coli* 1-176-05_S3_C2 (*E_coli_1*; A0A017IGS3), *Escherichia coli* DEC1A (*E_coli_D*; H4HXR7) and *Enterococcus gallinarum* (*E_gall*; U1EIW9) were compared to PduOC from *Salmonella enterica* (*S_ente*; A0A0F6B395). Amino acids forming the heme-binding pocket in PduOC are marked with a yellow background. His18 and the corresponding His residues in the other proteins are indicated with a red arrow. The GAMA sequence in PduOC is marked with a grey background. These four amino acids are located at the N-terminus of the recombinantly produced His-tag-free PduOC protein that was used for crystallization and biochemical assays. Numbering is such that residue 1 (Gly) is the first within PduOC.
